# CDK12 globally stimulates RNA polymerase II transcription elongation and carboxyl-terminal domain phosphorylation

**DOI:** 10.1093/nar/gkaa514

**Published:** 2020-06-17

**Authors:** Michael Tellier, Justyna Zaborowska, Livia Caizzi, Eusra Mohammad, Taras Velychko, Björn Schwalb, Ivan Ferrer-Vicens, Daniel Blears, Takayuki Nojima, Patrick Cramer, Shona Murphy

**Affiliations:** Sir William Dunn School of Pathology, University of Oxford, Oxford OX1 3RE, UK; Sir William Dunn School of Pathology, University of Oxford, Oxford OX1 3RE, UK; Max Planck Institute for Biophysical Chemistry, Department of Molecular Biology, Am Fassberg 11, 37077 Göttingen, Germany; Max Planck Institute for Biophysical Chemistry, Department of Molecular Biology, Am Fassberg 11, 37077 Göttingen, Germany; Max Planck Institute for Biophysical Chemistry, Department of Molecular Biology, Am Fassberg 11, 37077 Göttingen, Germany; Max Planck Institute for Biophysical Chemistry, Department of Molecular Biology, Am Fassberg 11, 37077 Göttingen, Germany; Sir William Dunn School of Pathology, University of Oxford, Oxford OX1 3RE, UK; Mechanisms of Transcription Laboratory, The Francis Crick Institute, 1 Midland Road, London NW1 1AT, UK; Sir William Dunn School of Pathology, University of Oxford, Oxford OX1 3RE, UK; Max Planck Institute for Biophysical Chemistry, Department of Molecular Biology, Am Fassberg 11, 37077 Göttingen, Germany; Sir William Dunn School of Pathology, University of Oxford, Oxford OX1 3RE, UK

## Abstract

Cyclin-dependent kinase 12 (CDK12) phosphorylates the carboxyl-terminal domain (CTD) of RNA polymerase II (pol II) but its roles in transcription beyond the expression of DNA damage response genes remain unclear. Here, we have used TT-seq and mNET-seq to monitor the direct effects of rapid CDK12 inhibition on transcription activity and CTD phosphorylation in human cells. CDK12 inhibition causes a genome-wide defect in transcription elongation and a global reduction of CTD Ser2 and Ser5 phosphorylation. The elongation defect is explained by the loss of the elongation factors LEO1 and CDC73, part of PAF1 complex, and SPT6 from the newly-elongating pol II. Our results indicate that CDK12 is a general activator of pol II transcription elongation and indicate that it targets both Ser2 and Ser5 residues of the pol II CTD.

## INTRODUCTION

The phosphorylation status of the C-terminal domain (CTD) of RNA polymerase II (pol II) plays a major role in expression of non-coding and protein-coding genes (1). In humans, the CTD comprises 52 repeats of the consensus heptapeptide Tyr1/Ser2/Pro3/Thr4/Ser5/Pro6/Ser7 and is reversibly phosphorylated by several kinases during transcription (1). CDK12 is the metazoan ortholog of the *Saccharomyces cerevisiae* CTD Ser2 kinase, Ctk1 (2,3). Knockdown of CDK12 results in loss of CTD Ser2 phosphorylation in *Drosophila*, *Caenorhabditis elegans* and human cells (3–10). In addition, treatment with the CDK12/CDK13 inhibitor THZ531 in Jurkat cells affects pol II CTD Ser2 phosphorylation in a concentration-dependent manner (7). Analog-sensitive (as) kinase technology has also been used to demonstrate rapid loss of Ser2 phosphorylation after inhibiting the *Schizosaccharomyces pombe* CDK12 homologue Lsk1 (11). However, knockdown of CDK12 in HCT116 cells or inhibition of CDK12^as^ in HeLa or HCT116 cells does not always cause loss of Ser2P (12,13), and detecting loss of Ser2P after CDK12 inhibition appears to depend on the anti-Ser2P antibody used (14). In addition, CDK12 mainly phosphorylates Ser5 *in vitro* (15,16).

CDK12 forms a complex with Cyclin K and is recruited to transcription units of pol II–transcribed genes (7,17–19) and enhancers (7). Blazek *et al.* have shown that depletion of CDK12 results in reduced expression of a small subset of genes (3.9%), enriched for genes involved in the DNA damage response (DDR), such as BRCA1 and ATR (4). Knockdown of Cyclin K/CDK12 complexes also causes elevated sensitivity to DNA damage inducing agents and accumulation of the DNA damage biomarker γ-H2AX (4). In addition, CDK12(−/−) embryos have reduced expression of genes involved in the DDR (20) and mutations that impair CDK12 kinase activity are associated with misregulation of expression of DNA repair genes and ovarian carcinoma (21,22). Downregulation of key DDR genes has also been observed by RNA-seq analysis following CDK12 depletion (12) and by microarray analysis after treatment of cells with THZ531 (7). In line with these findings, CDK12 is frequently mutated in high-grade serous ovarian carcinoma (HGSOC) (23). Interestingly, small molecule inhibitors of CDK12 also affect translation (24).

Pol II ChIP-seq and transient transcriptome sequencing (TT-seq) analyses suggest that elongation of transcription of genes sensitive to THZ531 is defective (7,25). In addition, it has been recently shown in mES cells, human tumours and mouse and human cell lines that downregulation of DDR genes following depletion of CDK12 or inhibition of CDK12/CDK13 results from the activation of intronic poly(A) sites, leading to the production of prematurely terminated gene products (10,25). However, the primary effect of selective inhibition of CDK12 activity on transcription remained unclear.

To better understand the function of the kinase activity of CDK12 in transcription, we have used CRISPR/Cas9 gene engineering to mutate the endogenous CDK12 gene in HEK293 cells to produce an analog-sensitive CDK12 (CDK12^as^) that can be selectively inhibited by bulky ATP analogs (26). We have combined the use of this CDK12^as^ cell line with two complementary functional genomics methods: TT-seq (27) and native elongating transcript sequencing for mammalian cells (mNET-seq) (28,29). TT-seq combines 4sU-metabolic labeling with RNA fragmentation to monitor synthesis of newly transcribed RNAs genome-wide. mNET-seq isolates and sequences the RNA in the active site of immunoprecipitated pol II to provide single-nucleotide resolution of pol II occupancy and CTD modification status.

We find that short-term inhibition of CDK12 causes a global decrease of pol II elongation velocity as measured by TT-seq and loss of phosphorylation of Ser2 and Ser5 of the pol II CTD as measured by mNET-seq and western blot. Elongation of transcription of the vast majority of pol II-transcribed genes is affected. CDK12 inhibition also causes the loss of key elongation factors from pol II transcription units, identifying CDK12 as a global regulator of pol II CTD Ser2 and Ser5 phosphorylation and transcription elongation in human cells.

## MATERIALS AND METHODS

### Cell culture

HEK293 cells were obtained from ATCC (ATCC^®^ CRL-1573™). HEK293 parental cells and CDK12^as^ HEK293 cells were grown in DMEM medium supplemented with 10% foetal calf serum, 100U/ml penicillin, 100 μg/ml streptomycin, 2 mM l-glutamine at 37°C and 5% CO_2_. HEK293 cells were treated with 7.5 μM 1-NM-PP1 (Cayman Chemical Company) for 15 and 30 min. As a negative control, HEK293 cells were treated with DMSO (the resuspension vehicle for NM). Cells were routinely checked to be free of mycoplasma contamination using Plasmo Test Mycoplasma Detection Kit (InvivoGen, rep-pt1).

### Analog sensitive cell line creation

Guide RNAs were computationally designed.

Guide RNA 1: GGTCCATATACTCAAATACAA

Guide RNA 2: GTATATGGACCATGACTTAAT

The modified CDK12 genomic sequence (RefSeq Accession NM_016507) (500 bp either site of the mutation) was cloned into pcDNA3 and used as the repair template for genome editing. The repair template contains a TTT to GGT mutation. The guide (g)RNAs inserts were cloned into the pX462 vector (obtained from Addgene). HEK293 cells were transfected with the gRNA vectors and correction template using Lipofectamine 2000 (Life Technologies) following the manufacturer instructions. Single clones were isolated by low density plating after Puromycin and Neomycin selection. Genomic DNA from each clone was analysed using PCR and Sanger sequencing.

### gDNA preparation

Cells were incubated in 180 μl ChIP lysis buffer for 10 min and sonicated for 3 min (30 s on/30 s off) using a Q800R2 sonicator (QSONICA). 20 μl ammonium acetate was added (final conc. 400 mM) and samples were mixed with 200 μl phenol chloroform by vortexing. The samples were centrifuged (13 000 g, 5 min, 25°C) and the upper phase was transferred to a new tube. Genomic DNA was precipitated in 70% ethanol, pelleted by centrifugation (13 000 g, 5 min, 25°C) and dissolved in nuclease free water. A CDK12 fragment was PCR amplified using the following primers: Forward 5′ CCAGCCATATGGGTGGCAGAGTGAGAGTCT 3′; Reverse 5′ CCAGCGCGGCCGCGCAGAAAAGGCCAATCA 3′. DNA was purified using a QIAquick PCR purification kit (Qiagen) and sequenced by the SourceBioscience Sanger Sequencing Service, Oxford.

### RNA preparation

RNA was extracted from HEK293 cells using TRIzol (Invitrogen) according to the manufacturer's instructions. Reverse-transcription (RT) was performed with 1 μg of RNA using random hexamers with the SuperScript III kit (Invitrogen) according to the manufacturer's instructions. Sequencing was performed on a cDNA PCR fragment generated with Forward primer: CGCTGTGTGGACAAGTTTGA and Reverse primer: GCTGGTGTGTAACGTTCCTC). Sequencing primer used: GCTTCCCAATCACAGCCATT.

### Cell proliferation analysis

Cells were seeded at 500/well in 95 μl in 96-well microplates (Greiner, 655090) and measured every 12 or 24 h by adding alamarBlue HS (Invitrogen) (1/20) and reading in a fluorimeter after 1 hour's incubation, according to the manufacturer's instructions. NM-PP1 or DMSO was added to the cells as noted on the figure. Continuous xCelligence analysis was carried out in 96-well E-Plates (H003957) after seeding the cells at 500/well and NM or DMSO was added as noted on the figure, 36 hours after seeding.

### Chromatin immunoprecipitation (ChIP)

ChIP analysis were performed as previously (30) using antibodies against control IgG (sc-2027, Santa Cruz), Pol II (MABI0601, MBL International), Ser2P (MABI0602, MBL International), Ser2P (ab5095, Abcam), Ser5P (MABI0603, MBL International), Ser5P (ab5131, Abcam), CPSF73 (A301-091A, Bethyl Laboratories), LEO1 (A300-175A, Bethyl Laboratories), CDC73 (A300-170A, Bethyl Laboratories), SPT6 (15616S, Cell Signaling Technology). ChIP samples were analysed by real-time qPCR using QuantiTect SYBR Green PCR kit (Qiagen) and Rotor-Gene RG-3000 (Corbett Research). Signals are presented as percentage of Input after removing the background signal from the IP with the IgG antibody. The sequence of primers used for qRT-PCR is given in Table 1.

**Table 1.** List of primers used in this study

Name	Sequence of forward primer	Sequence of reverse primer
NEG	TGGTACAACCACAGCTCAGTG	AAGCTGGACATGGTTGTGTG
*KPNB1* TSS+0.2	TTACTTCCTCCCTCCAAATGGG	ACAGCCTCCCTTCCTTCTTTC
*KPNB1* TSS+3.2	GCCCAGAGAACAAGAAATCG	GGAATGGACAAGCTGTGTTG
*KPNB1* TSS+20.2	TGCAAGAGCCAGTGGGAACACTT	CCTCTACTCAGCAATGATACTTC
*KPNB1* pA-4.8	CTGAGGAAACTGAAGAACCAAG	GAAGGCAGTGCTTGCCAGAAT
*KPNB1* pA-2.9	GAGGAGTGTGCACGGATGCTGAA	CCAAGATGGCCGATGTTATGG
*KPNB1* pA-0.4	TAGTTACCGTCTGCTTGGGAAGATG	CCTCTGACAGCAAGTCCAACATT
*KPNB1* pA+1.4	GACTCATCACACCAAGGTCAC	GATAGTGCTGGGAAGGAAATGG
*KPNB1* pA+2.6	GTACATCTCAGCTTTGGCATATG	GCCCAGAACATAGCAGGCATTGC
*KPNB1* pA+4.1	GTTTCACCGTGTTAGCCAGGATGG	CCACAGCCATGTTCATTTCTGC
*CCND2* TSS	AGAGCGAGACCAGTTTTAAGGG	TGGCTAAATAGGGGGTTTTCGG
*H2AFZ1*	ATGGGCGTTTTGTGTATGCG	CGACATCGAAACACGTGATTCC
*H1F0*	TGTGCAAGGACAGCAACAAC	GCCAACAAAACACACAAGCC
*HIST1H1E*	TAAACCAAAGACCGCCAAGC	AAGCAGTTGGCCAAAGGAAC
*HIST1H2AH*	AGTATGTCTGGACGTGGCAAG	TCGGCATAATTACCCTTGCG
*HIST1H1C*	ACTCTGGTGCAAACGAAAGG	TTAACCTTGGGCTTGGCTTC

ChIP-seq of pol II in HEK293 (one replicate) or CDK12^as^ (biological duplicates) cells treated with DMSO or 7.5 μM 1-NM-PP1 was performed with the antibody sc-899 X (Santa Cruz Biotechnology). ChIP-seq of Ser2P (ab5095, Abcam), Ser5P (ab5131, Abcam), CPSF73 (A301–091A, Bethyl Laboratories), LEO1 (A300-175A, Bethyl Laboratories), and SPT6 (15616S, Cell Signaling Technology) were performed in biological duplicates. Preparation of ChIP-seq library and ChIP sequencing was prepared with the NEBNext Ultra II DNA Library Prep Kit for Illumina (NEB), according to the manufacturer's instructions or conducted by the high throughput genomics team of the Wellcome Trust Centre for Human Genetics (WTCHG), Oxford.

### Co-immunoprecipitation

For each sample: 100 μl of Dynabeads M-280 Sheep anti-mouse IgG (Thermo Fisher) were pre-blocked overnight at 4°C on a wheel in 1 ml of PBS supplemented with 0.5% BSA. The next day, the beads were washed three times in IP buffer (25 mM Tris–HCl pH 8.0, 150 mM NaCl, 0.5% NP-40, 10% Glycerol, 2.5 mM MgCl_2_), before being incubated for 2 hours at 4°C on a wheel in 600 μl of IP buffer supplemented with 5 μg of antibody and protease inhibitor cocktail (cOmplete™, EDTA-free Protease Inhibitor Cocktail, Sigma-Aldrich). In the meantime, a 70–80% confluent 15 cm dish of CDK12^as^ cells was washed twice with ice-cold PBS before being scrapped with ice-cold PBS supplemented with protease inhibitor cocktail. The cells were pelleted at 500 g for 15 min at 4°C. The pellets were re-suspended in 800 μl of Lysis buffer (50 mM Tris–HCl pH 8.0, 150 mM NaCl, 1% NP-40, 10% glycerol, 2.5 mM MgCl_2_, protease inhibitor cocktail, PhosSTOP (Sigma-Aldrich), 1× PMSF (Sigma-Aldrich), and 25–29 units of Benzonase (Merck Millipore)) and incubated at 4°C on a wheel for 30 min. After centrifuging for 15 min at 13 000 g at 4°C, 800 μl of Dilution buffer (150 mM NaCl, 10% glycerol, 2.5 mM MgCl_2_, protease inhibitor cocktail, PhosSTOP, and 1× PMSF) was added to the supernatant

The beads conjugated with antibodies were washed three times with IP buffer supplemented with protease inhibitor cocktail, then the beads and 1 mg of proteins were incubated at 4°C on a wheel for 2 h. The beads were then washed three times with IP buffer containing 150 or 300 mM NaCl and supplemented with protease inhibitor cocktail and three times with IP buffer without NP-40, containing 150 or 300 mM NaCl, and supplemented with protease inhibitor cocktail. Proteins were eluted in 40 μl of 1× LDS plus 100 mM DTT for 10 min at 70°C. Western blots were performed with NuPAGE Novex 3–8% Tris-Acetate Protein Gels (Life Technologies).

The following antibody was used for immunoprecipitation: total pol II (MABI0601, MBL International).

### Western blot

Western blot analysis was performed on chromatin and nucleoplasm extracts as previously described (29) until purification of the chromatin fraction. The chromatin pellet was digested in 100 μl of nuclease-free water supplemented with 1 μl of Benzonase (25–29 units, Merck Millipore) for 10 min at 37°C in a thermomixer (1400 rpm). 10 μg of proteins were boiled in 1× LDS plus 100 mM DTT. Western blots were performed with NuPAGE Novex 4–12% Bis–Tris Protein Gels (Life Technologies).

For whole cell extract, cells were washed in ice-cold PBS twice, collected in ice-cold PBS by 3000 rpm centrifuge for 5 min at 4°C. The pellets were re-suspended in RIPA buffer, kept on ice for 30 min with a vortexing step every 10 min. After centrifugation at 14 000 g for 15 min at 4°C, the supernatants were kept and quantified with the Bradford method. 20 μg of proteins were boiled in 1× LDS plus 100 mM DTT. Western blots were performed with NuPAGE Novex 4–12% Bis–Tris Protein Gels (Life Technologies).

The following antibodies were used: Pol II (MABI0601, MBL International), Ser2P (MABI0602, MBL International), Ser2P (5095, Abcam), Ser5P (MABI0603, MBL International), Ser5P (5131, Abcam), LEO1 (A300–175A, Bethyl Laboratories), CDC73 (A300–170A, Bethyl Laboratories), SPT6 (15616S, Cell Signaling Technology), CDK12 (11973S, Cell Signaling Technology), Cyclin K G-11 (sc-376371, Santa Cruz Biotechnology), β-tubulin (6046, Abcam), nucleolin (ab22758, Abcam), and histone H3 (ab1791, Abcam). Secondary antibodies were purchased from Merck Millipore (Goat Anti-Rabbit IgG Antibody, HRP-conjugate, 12-348, and Goat Anti-Mouse IgG Antibody, HRP conjugate, 12-349), the chemiluminescent substrate (SuperSignal West Pico PLUS) from Thermo Fisher, and the membranes visualized on an iBright FL1000 Imaging System (Thermo Fisher). Quantification of the western blots was performed with Image Studio Lite software.

### mNET-seq and library preparation

mNET-seq was carried out in biological duplicates as previously described (29) with minor changes. In brief, the chromatin fraction was isolated from 4 × 10^7^ CDK12^as^ cells treated with DMSO and 1-NM-PP1. Chromatin was digested in 100 μl of MNase (40 units/μl) reaction buffer for 2–3 min at 37°C in a thermomixer (1400 rpm). After addition of 10 μl EGTA (25mM) to inactivate MNase, soluble digested chromatin was collected by 13 000 rpm centrifuge for 5 min. The supernatant was diluted with 400 μl of NET-2 buffer and antibody-conjugated beads were added. Antibodies used: Pol II (MABI0601, MBL International), Ser2P (MABI0602, MBL International), Ser2P (5095, Abcam), and Ser5P (5131, Abcam). Immunoprecipitation was performed at 4°C for 1 h. The beads were washed with 1 ml of NET-2 buffer six times with 100 μl of 1xPNKT (1xPNK buffer and 0.05% Triton X-100) buffer once in cold room. Washed beads were incubated in 200 μl PNK reaction mix in Thermomixer (1400 rpm) at 37°C for 6 min. After the reaction, beads were washed with 1 ml of NET-2 buffer once and RNA was extracted with Trizol reagent. RNA was suspended in urea Dye and resolved on 6% TBU gel (Invitrogen) at 200 V for 5 min. In order to size select 35–100 nt RNAs, a gel fragment was cut between BPB and XC dye markers. A 0.5 mlL tube was prepared with 3–4 small holes made with 25G needle and placed in a 1.5 ml tube. Gel fragments were placed in the layered tube and broken down by centrifugation at 12 000 rpm for 1 min. The small RNAs were eluted from the gel using RNA elution buffer (1 M NaOAc and 1 mM EDTA) at 25°C for 1 h in Thermomixer (900 rpm). Eluted RNA was purified with SpinX column (Coster) with two glass filters (Millipore) and the flow-through RNA was ethanol precipitated. RNA libraries were prepared according to manual of NEBNext small RNA library prep kit (NEB). 12–14 cycles of PCR were used to amplify the library. Deep sequencing (Hiseq4000, Illumina) was conducted by the high throughput genomics team of the Wellcome Trust Centre for Human Genetics (WTCHG), Oxford.

### TT-seq and library preparation

TT-seq was performed as described (27,31), with minor changes. Specifically, 6 × 10^7^ cells, grown in absence of penicillin and streptomycin, from two biological replicates were treated for 15 and 30 min with solvent (DMSO) or 7.5μM 1-NM-PP1 (NM) at 37°C and 5% CO_2_. For the 15 min treatment, cells were exposed to 10 min 500 μM of 4-thiouracil (4sU, Carbosynth, NT06186) after 5 min of DMSO or NM treatment (15 min total treatment). For the 30 min treatment, cells were exposed to 10 min 500 μM of 4-thiouracil (4sU, Carbosynth, NT06186) after 20 min of DMSO or NM treatment (30 min total treatment). Cells were lysed in 5 ml of QIAzol (Qiagen) and 300 ng of RNA spike-ins mix were added to each sample. RNA spike-ins were produced as described (27). RNAs were sonicated to obtain fragments of <10 kb using AFAmicro tubes in a S220 Focused-ultrasonicator (Covaris Inc, parameters: 10 s, peak power 100, cycles 200, duty cycle 1%). 4sU-labeled RNAs were purified from 240 μg of each of the fragmented RNAs. Biotinylation and purification of 4sU-labeled RNAs was performed as described (27,32). 100 ng of input RNA was used for strand-specific library preparation according to the Ovation Universal RNA-seq System (NuGEN). Libraries were prepared using random hexamer priming only.

## QUANTIFICATION AND STATISTICAL ANALYSIS

### TT-seq data preprocessing and global normalization parameters

Paired-end 75 base reads with additional 6 base reads of barcodes were obtained for each of the samples. Reads were demultiplexed and mapped using STAR 2.5.2b (33) to the hg20/hg38 (GRCh38) genome assembly (Human Genome Reference Consortium) with maximum 2% mismatches and only unique alignments were retained. SAMtools was used to quality filter SAM files, whereby alignments with MAPQ smaller than 7 (-q 7) were skipped and only proper pairs (-f2) were selected. Further data processing was carried out using the R/Bioconductor environment. We used a spike-in (RNAs) normalization strategy essentially as described (27) to allow observation of global shifts (sequencing depth) ${\sigma _j}$, cross-contamination rate ${\epsilon _j}$ (proportion of unlabeled reads purified in the TT-seq samples) and antisense bias ratio ${c_j}$ (ratio of spurious reads originating from the opposite strand introduced by the reverse transcription reaction). Read counts (*k_ij_*) for spike-ins were calculated using HTSeq (34). Calculations for each parameter are described in the following in more detail.

### Antisense bias ratio ${c_j}$

Antisense bias ratios were calculated for each sample *j* according to

$$\begin{equation*}\ {c_j} = \mathop {{\rm{median}}}\limits_i \left( {\frac{{k_{ij}^{antisense}}}{{k_{ij}^{sense}}}} \right)\ \end{equation*}$$

for all available spike-ins *i*.

### Cross-contamination rate ${\epsilon _j}$

The cross-contamination rate ${\epsilon _j}$ was calculated for each sample *j* as

$$\begin{equation*}{\epsilon _j}{\rm{\ }} = \mathop {{\rm{median}}}\limits_i \left( {\frac{{{k_{ij}}}}{{{l_i}}}} \right)/{\sigma _j}\ \end{equation*}$$

using the unlabeled spike-ins *i* for TT-seq samples.

### Definition of transcription units based on the UCSC RefSeq genome assembly GRCh38 (RefSeq-TUs)

For each annotated gene, transcription units were defined as the union of all existing inherent transcript isoforms (UCSC RefSeq GRCh38).

### TT-seq processing with global normalization parameters

The number of transcribed bases (*tb_j_*) or read counts (*k_j_*) for RefSeq-TUs were normalized and corrected for antisense bias *c_j_*, sequencing depth ${\sigma _j}$ as follows using the parameters calculated as described above. The cross-contamination rate estimates for TT-seq in HEK293 were very low and were thus not corrected for.

### Antisense bias correction

The real number of read counts or coverage *s_ij_* for transcribed unit *i* in sample *j* was calculated as

$$\begin{equation*}\ {s_{ij}} = \frac{{{S_{ij}} - {c_j}{A_{ij}}}}{{1 - c_j^2}}\ \end{equation*}$$

where *S_ij_* and *A_ij_* are the observed numbers of read counts or coverage on the sense and antisense strand.

### Read counts per kilobase (RPK)

RPKs were calculated upon antisense bias corrected read counts (*k_j_*) falling into the region of a RefSeq-TU divided by its length in kilobases.

### TT-seq normalization

For a robust normalization, we first identified a subgroup of RefSeq-TU’s as non-differentially expressed (non-DE) for both time points over the response of NM-PP1(CDK12^as^ inhibitor) using DESeq2 package. Based on the antisense bias corrected RPKs, a group of expressed TUs [n = 5631] was defined to comprise all TUs with an RPK of 100 or higher in two summarized replicates of TT-seq 15 min samples without inhibitor treatment. An RPK of 100 corresponds to approximately a coverage of 10 per sample due to an average fragment size of 200. Within this group, a subset of TUs having a length >50 kb [*n* = 1867] was used to identify the non-DE TU’s.

The antisense bias corrected coverage *s_ij_* of transcribed unit *i* in sample *j* was normalized for sequencing depth as

$$\begin{equation*}\ {t_{ij}} = \frac{{s_{ij}^{{\rm{TT}} - {\rm{seq}}}}}{{\sigma _j^{{\rm{TT}} - {\rm{seq}}}}}\ \end{equation*}$$

where ${\sigma _j}$ was balanced between replicates via classical size factor normalization to gain statistical power in the differential expression analysis.

On the resulting 140 non-DE RefSeq-TUs, size factors for each sample were then estimated as described by Equation (5) in DESeq package (34) to correct for library size and sequencing depth variations.

### Visualization

For visualization, read coverages were summed up over replicates, size factors for each condition were calculated on the 140 non-DE RefSeq-TUs by Equation (5) in DESeq package (34) and used to correct for library size and sequencing depth variation.

For plotting, the expressed gene set is defined as follows: Based on the antisense bias corrected RPKs, a group of expressed TUs [*n* = 11 282] was defined to comprise all TUs with an RPK of 20 or higher in two summarized replicates of TT-seq 15 min samples without inhibitor treatment. An RPK of 20 corresponds to approximately a coverage of 2 per sample due to an average fragment size of 200.

### Simulation of TT-seq data based on elongation velocity profiles

Based on the following model we simulated TT-seq coverage values by providing elongation velocity profiles $v( t )$, a labeling duration ${t^{lab}}$ and a uracil content dependent labeling bias

$$\begin{equation*}\ {l_f} = \ 1 - {\left( {1 - {p^{lab}}} \right)^{\# {u_f}}}\end{equation*}$$

${p^{lab}}$ denotes the labeling probability (set to 0.01) and $\# {u_f}$ the number of uracil residues of a given fragment $f$ (set to 0.28 times fragment length). Elongating polymerases are propagated at nucleotide resolution along the template in a time-dependent manner. Each position of elongating polymerases ${\tau ^*}$ can be mapped to its respective time of transcription ${t^*}$with an elongation velocity profile $v( t )$ as

$$\begin{equation*} {\tau ^*} = \mathop \int \limits_0^{{t^*}} v\left( t \right)dt \end{equation*}$$

Consequently, the progression of a polymerase at any given position ${\tau ^*}$ with velocity $v( {{t^*}} )$for time ${t_p}$can be calculated as

$$\begin{equation*}{\tau _{{t_p}}} \left( t \right) = \mathop \int \limits_0^{{t_p} + t*} v\left( t \right)dt - \mathop \int \limits_0^{{t^*}} v\left( t \right)dt = \mathop \int \limits_{t*}^{{t_p} + t*} v\left( t \right)dt\ \end{equation*}$$

while $v( t )$ is the current velocity function at absolute time.

With this equation, the movement of single polymerases over the course of labeling can be tracked. Start and end positions of fragments can be derived given the positions of any given polymerase at$\ \tau ( {t,t - {t^{lab}}} )$ for any given transcription time. We used the number of uracil residues present in the RNA fragment $\# {u_f}$ to weight the amount of coverage contributed by this fragment as ${l_f}$. Additionally, we applied a size selection similar to that in the original protocol for fragments below 80 bp in length with a sigmoidal curve that mimics a typical size selection spread. This was done for templates resembling genes of sizes ∼0,1- 2000 kbp. The resulting gene-wise RNA synthesis profiles were subsequently accumulated to yield meta-gene profiles.

### mNET-seq data processing

mNET-seq data were processed as follows: adapters were trimmed with Cutadapt in paired-end mode with the following parameters: -q 15, 10 **–**minimum-length 10 –A GATCGTCGGACTGTAGAACTCTGAAC –a AGATCGGAAGAGCACACGTCTGAACTCCAGTCAC. Trimmed reads were mapped to the human hg38 reference sequence with Tophat2 and the parameters –g 1 –r 3000 –no-coverage-search. SAMtools was used to retain only properly paired and mapped reads (-f 3). A custom python script (28) was used to obtain the 3′ nucleotide of the second read and the strandedness of the first read. Strand-specific bam files were generated with SAMtools. FPKM normalized bigwig files were generated for each bam files with Deeptools2 (35) bamCoverage tool (-bs 1 –p max –normalizeUsing RPKM). For plotting, the expressed gene set is defined as follows: Based on the antisense bias corrected RPKs, a group of expressed TUs [n = 11282].

### mNET-seq and ChIP-seq normalization

The total pol II mNET-seq treated with DMSO and NM 15 min were re-normalized to a set of 140 genes found to be non-affected in the spiked-in TT-seq analysis after 15 and 30 min inhibition. The re-normalization factor was calculated from the average fold change on total pol II signal between DMSO and the NM conditions across the gene bodies of this set of non-affected genes found in TT-seq. For the total pol II mNET-seq metaprofiles and total pol II quantification, the re-normalization factors for NM 15 min R1 and R2 are 0.90 and 1.11, respectively. The Ser2P and Ser5P 15 min were normalized to the CTD phosphorylation signals in histone genes (see Results and Supplementary Figure S4) with the following normalization factors for the merged biological replicates: Ser2P MABI0602: 0.626, Ser2P ab5095: 0.544 and Ser5P ab5131: 0.791.

### ChIP-seq data processing

Adapters were trimmed with Cutadapt in paired-end mode with the same parameters as mNET-seq. Obtained sequences were mapped to the human hg38 reference genome with Bowtie2. Properly paired and mapped reads were filtered with SAMtools. PCR duplicates were removed with Picard MarkDuplicates tool. FPKM normalized bigwig files were generated for each bam files with Deeptools2 bamCoverage tool (-bs 10 –p max –normalizeUsing RPKM –e). For the HEK293 ChIP-seq presented in Supplementary Figure S2B, the pol II signal was normalized to the *KPNB1* gene body (TSS + 500 bp to poly(A) site, NM normalization factor: 0.494) as this gene was found to be non-affected by pol II ChIP-qPCR. CDK12as pol II ChIP-seq NM normalization factors are 1.345 and 0.840 for replicates 1 and 2, respectively. Ser2P NM normalization factors are 1 and 1.429 for replicates 1 and 2, respectively. Ser5P NM normalization factors are 1.275 and 1.269 for replicates 1 and 2, respectively.

### Metagene profiles

Metagene profiles of genes scaled to the same length were then generated with Deeptools2 computeMatrix tool with a bin size of 10 bp and the plotting data obtained with plotProfile –outFileNameData tool. Graphs representing the (IP – Input) signal (ChIP-seq) or the mNET-seq signal were then created with GraphPad Prism 8.4.0. Metagene profiles are shown as the average of two biological replicates.

### *P*-values and significance tests

*P*-values were computed with an unpaired two-tailed Student's *t* test. Statistical tests were performed in GraphPad Prism 8.4.0.

## RESULTS

### Rapid and specific CDK12^as^ inhibition in human cells

Several studies using RNAi-mediated knockdown of CDK12 have highlighted roles for this kinase in CTD phosphorylation and expression of DDR genes (3,4,17). To assess whether these effects are mediated by CDK12 kinase activity, the loss of CDK12 itself, or indirect effects due to the long time-frame of knockdown, we produced a CDK12^as^ HEK293 cell line, using CRISPR/Cas9, where the gatekeeper phenylalanine residue (codon TTT) is mutated to glycine (codon GGT) (Supplementary Figure S1A). This allows rapid and selective inhibition of CDK12 kinase activity with a bulky ATP-analog 1-NM-PP1 (NM) (14,16,36), without affecting levels of the protein itself, which has been shown to interact with other components of the transcription/RNA processing machinery (12,19,37). Importantly, the growth rate of the CDK12^as^ cells is not lower than that of the parental HEK293 cell line (Supplementary Figure S1B). Also, CDK12 and Cyclin K protein levels are unchanged in CDK12^as^ cells compared to the parental cell line, as measured by western blot analyses (Supplementary Figure S1C and D).

To determine which concentration of NM to use, we followed the growth of CDK12^as^ and the parental HEK293 cells after the addition of 5, 7.5 and 10 μM of NM to the medium using either an alamarBlue HS cell viability assay or continuous label-free monitoring by xCelligence (Supplementary Figure S1E and F). In both cases, 7.5 μM NM affects the viability and growth of CDK12^as^ cells with less effect on HEK293, whereas 10 μM NM affects both cell lines. This is in agreement with previously published data on CDK12^as^ inhibition in HeLa cells (14). Importantly, short-term treatment of HEK293 and CDK12^as^ cells with 7.5 μM NM does not affect the protein levels of CDK12 and Cyclin K (Supplementary Figure S1G and H) and takes many hours to affect cell viability (Supplementary Figure S1E). We have therefore treated cells with this inhibitor concentration for 15 or 30 min, in order to determine the immediate effect of CDK12 inhibition. Importantly, pol II distribution on expressed transcription units in the parental cells is not affected by short-term NM treatment as measured by pol II chromatin immunoprecipitation followed by quantitative PCR (ChIP-qPCR) of *KPNB1* and ChIP-sequencing (ChIP-seq) (Supplementary Figure S2A and B).

### CDK12^as^ inhibition globally decreases RNA synthesis

**Figure 1.**
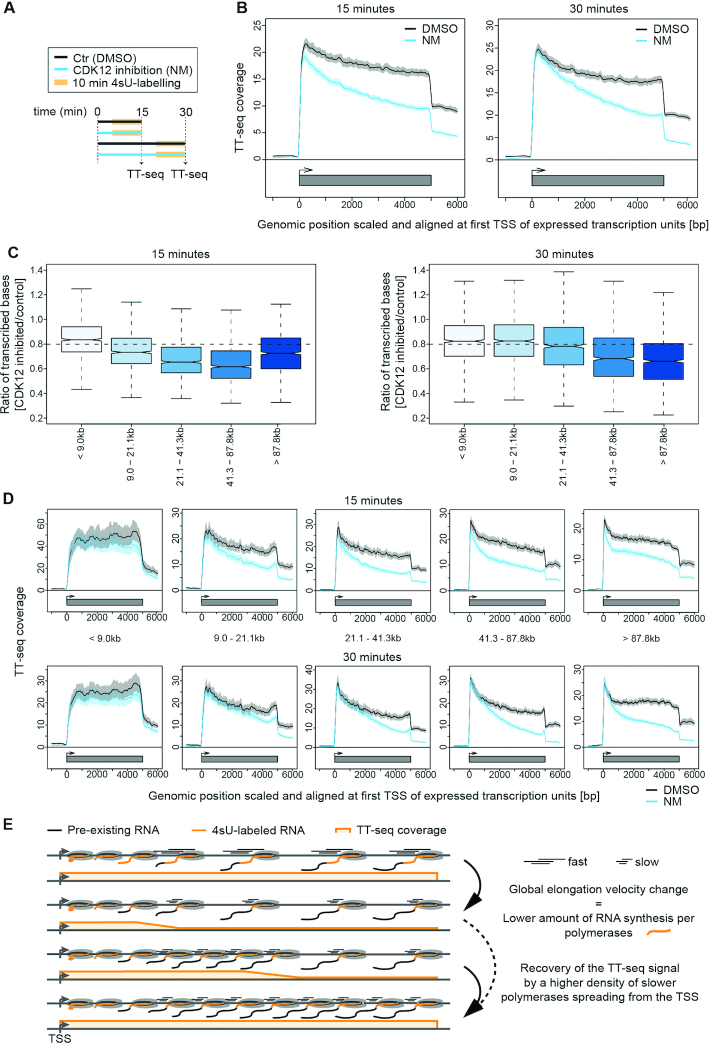
CDK12^as^ inhibition globally decreases RNA synthesis. (**A**) Experimental design. (**B**) Metagene analysis of TT-seq signal for expressed genes after treatment of cells with DMSO (black) versus 7.5 μM NM treatment (blue) for 15 min (left) or 30 min (right). The TT-seq coverage was averaged and aligned at their transcription start sites (TSSs) and polyadenylation (pA)-sites. Shaded areas around the average signal (solid lines) indicate confidential intervals. (**C**) Box plots of different length classes show the ratio of transcribed bases after 15 min (left) and 30 min (right) inhibition of CDK12^as^ compared to control. (**D**) Metagene analysis for different length classes comparing the average TT-seq signal before (DMSO treatment, black) and after CDK12^as^ inhibition (7.5 μM NM treatment, blue) for 15 min (upper panel) and 30 min (lower panel). The TT-seq coverage was averaged and aligned at their transcription start sites (TSSs) and polyadenylation (pA)-sites. Shaded areas around the average signal (solid lines) indicate confidential intervals. (**E**) Schematic representation of TT-seq signal changes along the gene body upon elongation velocity change. Upper panel: steady state transcription. Lower panels show TT-seq signal recovery spreading from the TSS.

To monitor changes in RNA synthesis upon CDK12^as^ inhibition, we performed TT-seq with RNA spike-ins after 15 and 30 min of NM treatment (Figure 1A). Also using this analysis, treatment with NM has little effect on transcription in the parental cells (Supplementary Figure S2C and D). However, metagene profiles of TT-seq signals averaged over expressed genes (see Materials and Methods) in CDK12^as^ cells show a decreased TT-seq signal across gene bodies after 15 min of inhibition, indicating reduced RNA synthesis, either as the result of reduced elongation or loss of polymerase, in 11 182 transcription units, including 10,393 protein-coding genes and 789 unclassified transcription units (Figure 1B–D). CDK12^as^ inhibition for 30 min instead leads to a recovery of RNA synthesis activity at the beginning of genes (Figure 1C, right, D lower panel).

Recovery of the TT-seq signal after 30 min could be explained by a higher density of slower polymerases spreading from the transcription start site and gradually populating the gene body (Figure 1E). This effect would not require any changes in initiation frequencies, but could stem only from an increase in polymerase density over genes due to slower elongation. After CDK12^as^ inhibition, polymerases dramatically slow down, resulting in a lower amount of RNA synthesis, which may be successively restored by a higher number of polymerases occupying the gene body provided the same number of initiation events. This model predicts that recovery of RNA synthesis takes longer for long genes and shorter for short genes (Figure 1C–E). As human genes take on average longer than 30 min to be transcribed, recovery of RNA synthesis and TT-seq signal would be limited to the 5′-region of genes and not observed for many 3′-regions, leading to the observed slope in the TT-seq metagene profile (Figure 1C–E).

To test this model further, we simulated TT-seq metagene profiles based on a kinetic model that computes RNA synthesis at each gene position based on initiation rates and elongation velocities. The model readily recapitulates the observed TT-seq profiles if we assume that CDK12 inhibition induces rapid and global downregulation of RNA elongation without affecting transcription initiation frequency (Supplementary Figure S2E and F). Thus, TT-seq analysis and kinetic modeling supports the notion that CDK12 is required for normal transcription elongation, and the elongation defect resulting from its inactivation is readily observed after rapid inhibition followed by immediate monitoring of RNA synthesis.

### Inhibition of CDK12^as^ affects transcription elongation

**Figure 2.**
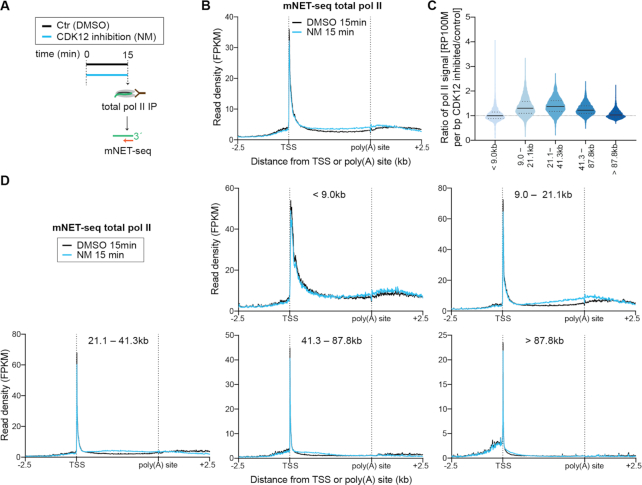
Inhibition of CDK12^as^ affects transcription elongation. (**A**) Experimental design. mNET-seq using an antibody against total pol II was performed in CDK12^as^ HEK293 cells after treatment with 15 min of DMSO solvent control or 7.5 μM NM. (**B**) Metagene analysis comparing the average mNET-seq signal before and after CDK12^as^ inhibition for 15 min of expressed genes. (**C**) Violin plots of the different length classes noted show the ratio of mNET-seq signal after 15 min inhibition of CDK12^as^ compared to control. (**D**) Metagene analysis for the different length classes noted comparing the average mNET-seq signal before and after CDK12^as^ inhibition for 15 min. The mNET-seq reads were averaged and aligned at their TSSs and (pA)-sites.

To confirm that the observed decrease in RNA synthesis activity results from reduced elongation, we performed mNET-seq of total pol II in CDK12^as^ cells after 15 min of NM treatment (Figure 2A). mNET-seq identifies the last nucleotide transcribed by sequencing the RNA present in the active site of immunoprecipitated pol II (29). The mNET-seq data were normalized to a set of genes found to be unaffected in TT-seq (see Materials and Methods). Inhibition of CDK12^as^ results in an increase of pol II signal in the gene body of the vast majority of expressed pol II-transcribed genes within 15 min of inhibition (Figure 2B and C), consistent with higher densities of more slowly-elongating pol II. ChIP-seq of pol II gives a similar picture after 15 min inhibition of CDK12^as^ (Supplementary Figure S3A). We have confirmed this increase of pol II occupancy in the gene body of a model gene, *KPNB1*, by performing pol II ChIP-qPCR after 15 and 30 min treatment of CDK12^as^ cells with NM (Supplementary Figure S3B). The higher pol II signal results from an increased pol II residence time in gene bodies due to slower elongation, and not from an increased amount of pol II entering elongation, as shown by the decrease in RNA synthesis in TT-seq (Figure 1).

Further analysis shows that transcription of pol II-transcribed genes of all lengths is affected by CDK12^as^ inhibition, with a wave of elongation-compromised pol II reaching the ends of the shortest genes first (Figure 2D). Genes <9 kb have a less pronounced elongation defect after CDK12^as^ inhibition than genes >9 kb as measured by both TT-seq and mNET-seq (Figures 1D and 2D). In addition, the elongation defect increases with increasing distance from the TSS for all gene length classes (Figure 1D). This is more apparent after analysis of TT-seq and mNET-seq data for the first few kilobases of genes >6.5 kb (Supplementary Figure S3C and D). Thus, the effect of CDK12^as^ inhibition starts early in the transcription cycle and increases with increasing distance from the TSS (Supplementary Figure S3C and D). Many of the genes <9 kb will therefore be too small to exhibit a major effect of CDK12^as^ inhibition on elongation as pol II will reach the end of genes soon after the elongation defect starts to occur.

Interestingly, on genes longer than 21.1 kb, pol II increases in the gene body but is specifically reduced downstream of the poly(A) site (Figure 2D), before the wave of slower polymerases reaches this region. Single gene examples of this phenomenon are shown in Supplementary Figure S3E and this can also be observed by pol II ChIP-qPCR of the *KPNB1* gene (Supplementary Figure S3B).

Since transcription of intron-containing protein-coding genes is affected by short-term inhibition of CDK12^as^, we investigated whether transcription of intronless (*n* = 5396), histone (*n* = 118), and snRNAs (*n* = 36) genes is also altered (Supplementary Figure S4A and B). TT-seq signals for intronless and pol II-transcribed snRNA genes decrease after 15 or 30 min inhibition, whereas mNET-seq signals on intronless genes are not markedly affected and slightly reduced on snRNA genes. We interpret this to mean that pol II is slower on these genes but pol II is not building up as they are relatively short (the intronless and snRNAs have an average size of 948 and 150 bp, respectively). TT-seq signals for histone genes instead are either unaffected or slightly increased and mNET-seq signals unaffected by CDK12^as^ inhibition. We therefore conclude that transcription of histone genes does not require CDK12 activity. We have confirmed this by pol II ChIP-qPCR for five different histone genes (Supplementary Figure S4C).

The primary effects of CDK12^as^ inhibition on transcription of intron-containing protein-coding genes are therefore an elongation defect in gene bodies and loss of pol II downstream of the poly(A) site. Taken together, the results indicate that CDK12 plays a role in maintaining efficient transcription elongation velocity and processivity on these human genes. Efficient transcription of intronless genes and snRNA genes also appears to require CDK12, although the defects are less apparent. Histone genes, on the other hand, are transcribed efficiently in the absence of CDK12.

### CDK12 phosphorylates transcribing pol II

**Figure 3.**
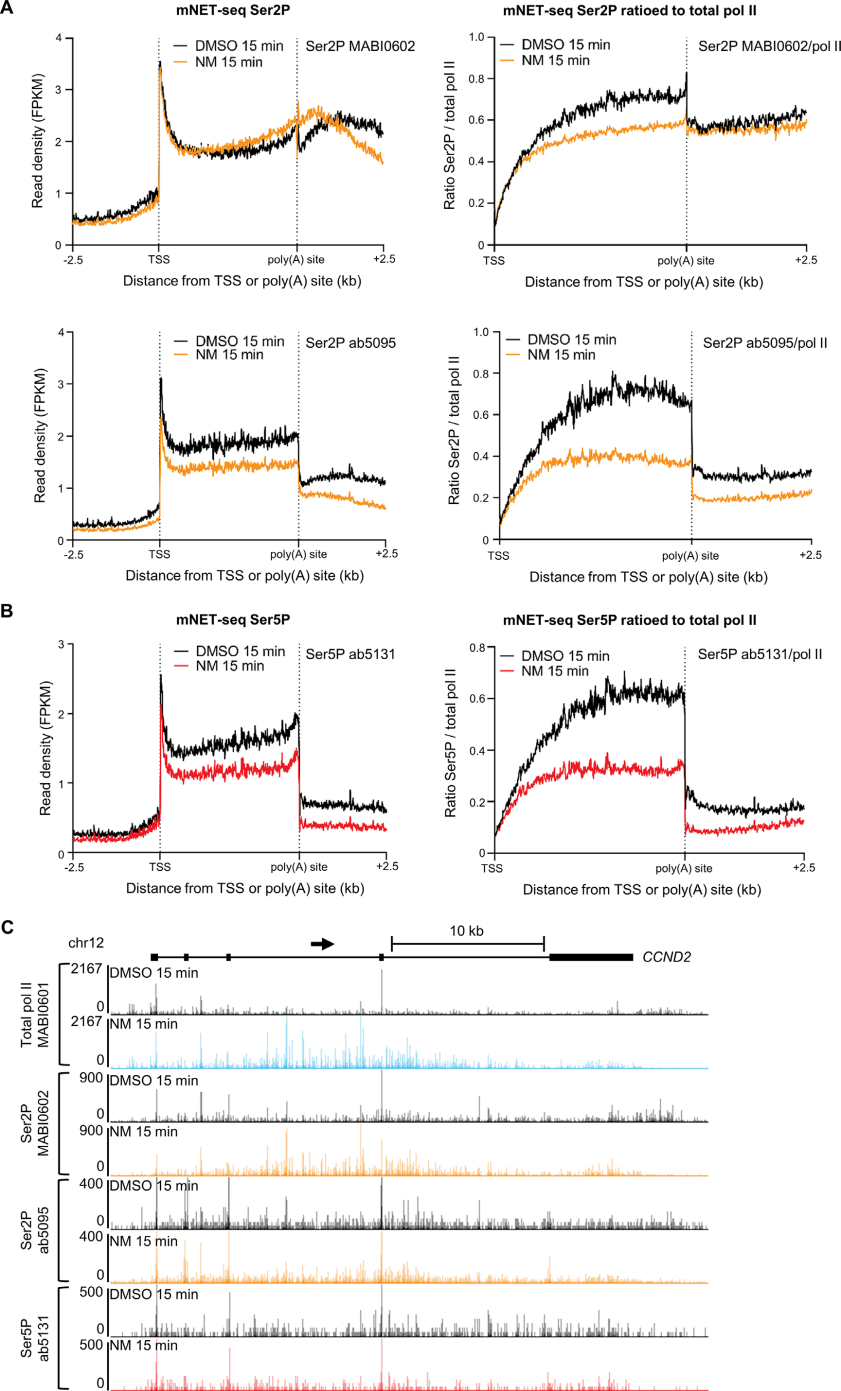
CDK12 phosphorylates transcribing pol II. (**A**) Meta-analyses of scaled expressed genes of mNET-seq for Ser2P with and without normalization to pol II after treatment of CDK12^as^ cells with DMSO (black) or 7.5μM NM (orange) for 15 min. (**B**) Meta-analyses of scaled expressed genes of mNET-seq for Ser5P with and without normalization to pol II after treatment of CDK12^as^ cells with DMSO (black) or 7.5 μM NM (red) for 15 min. (**C**) mNET-seq profiles across *CCND2* using a total pol II antibody, two Ser2P antibodies and one Ser5P antibody.

mNET-seq can also be carried out with CTD phospho-site-specific antibodies to detect nascent RNA associated with particular pol II phospho-isoforms (29). Accordingly, we have used mNET-seq to investigate the primary effect of CDK12^as^ inhibition on CTD phosphorylation. As short-term inhibition of CDK12^as^ is sufficient to cause global changes in transcription (Figures 1 and 2), we treated CDK12^as^ with NM for 15 min and obtained mNET-seq profiles for pol II CTD phosphorylation at Ser2P and Ser5P using antibodies against each of these phospho-forms (Figure 3).

**Figure 4.**
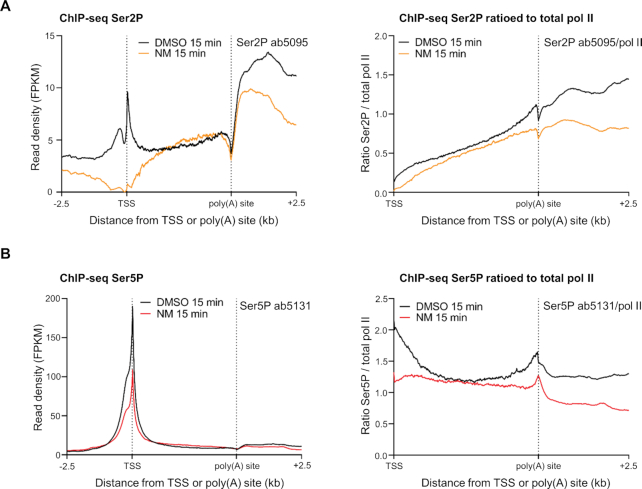
ChIP-seq analysis of CDK12 phosphorylation of transcribing pol II. (**A**) Meta-analyses of scaled expressed genes of ChIP-seq for Ser2P (ab5095) with and without normalization to pol II after treatment of CDK12^as^ cells with DMSO (black) or 7.5μM NM (orange) for 15 min. (**B**) Meta-analyses of scaled expressed genes of ChIP-seq for Ser5P (ab5131) with and without normalization to pol II after treatment of CDK12^as^ cells with DMSO (black) or 7.5 μM NM (red) for 15 min.

As transcription of histone genes is relatively unaffected by CDK12^as^ inhibition, we have investigated whether pol II CTD Ser2P and Ser5P levels are affected by CDK12^as^ inhibition. Ser2P and Ser5P ChIP-qPCR for five different histone genes indicates that Ser2P and Ser5P, ratioed and unratioed to pol II levels, is unaffected by NM treatment (Supplementary Figure S4C), indicating no requirement for CDK12 for CTD phosphorylation during transcription of these genes. Interestingly, the level of Ser2P relative to pol II on histone is generally much lower than on other protein-coding genes (a ratio of up to 0.6 on histone genes and a ratio of >40 at the end of *KPNB1* with ab5095) (Supplementary Figures S4 and S5), which is consistent with the relative lack of requirement for a Ser2 kinase. We have therefore used the levels of Ser2P and Ser5P on histone genes to normalize our mNET-seq Ser2P and Ser5P data (see Materials and Methods). We have displayed the results with and without normalisation of the mNET-seq signals to total pol II levels to take changes of pol II occupancy into account (Figure 3A and B). Figure 3C shows the genome browser track of the unratioed mNET-seq results for the *CCND2* gene, a ∼32 kb long gene displaying increased pol II signal in the gene body and a loss of pol II downstream of the poly(A) site. The mNET-seq signals for two different Ser2P antibodies and for the Ser5P antibody show a decrease after CDK12^as^ inhibition when ratioed to the total pol II level (Figure 3A and B). The same effect of CDK12^as^ inhibition on Ser2P and Ser5P phosphorylation is seen using ChIP-seq of Ser2P and Ser5P and ChIP-qPCR on *KPNB1* (Figure 4A, B, Supplementary Figure S5A and B) Interestingly, we observed a loss of Ser5P phosphorylation at the TSS of genes after CDK12^as^ inhibition, indicating that CDK12 acts as a Ser5 kinase at this step and could affect mRNA capping, which relies on Ser5P (38) (Supplementary Figure S5B). We first confirmed the loss of Ser5P by ChIP-qPCR on the TSS of *KPNB1* and *CCND2* after 15- and 30-min inhibition of CDK12^as^ (Supplementary Figure S5C and D). To investigate a possible effect on mRNA capping, we performed ChIP-qPCR of RNGTT, the enzyme responsible for the first two catalytic steps of the cap formation, and found a decrease in signal at the TSS following CDK12^as^ inhibition consistent with the reduction in Ser5P relative to total pol II (Supplementary Figure S5C and D).

We also carried out western blot analyses on chromatin-associated pol II with antibodies against different pol II CTD phospho-isoforms after treating cells with NM for 15 and 30 min (Supplementary Figure S6A and B). In line with the results of mNET-seq analysis (Figure 3A and B), inhibition of CDK12^as^ markedly affects Ser2 and Ser5 phosphorylation within 15 min, as measured with two different antibodies against these modifications (Supplementary Figure S6A and B). Both Ser2P and Ser5P continue to be affected after 30 min, with a further decrease in phosphorylation observed for Ser2P MABI0602 and Ser5P MABI0603 (Supplementary Figure S6A and B). In contrast, no loss of Ser2P or Ser5P occurs when HEK293 cells are treated with NM for 15 or 30 min (Supplementary Figure S6C and D). Loss of Ser2P and Ser5P after 15 min of inhibition of CDK12^as^ was also confirmed by performing immunoprecipitation of total pol II followed by western blotting with a Ser2P (ab5095) and Ser5P (ab5131) antibody (Supplementary Figure S6E). These results are consistent with previous *in vitro* kinase assays and analysis of CDK12^as^ HeLa cells (14,15). Taken together, the western and mNET-seq analyses indicate that CDK12 contributes to phosphorylation of the CTD of engaged pol II with activity towards both Ser2 and Ser5.

### CDK12 activity is required for stable association of elongation and termination factors with chromatin

**Figure 5.**
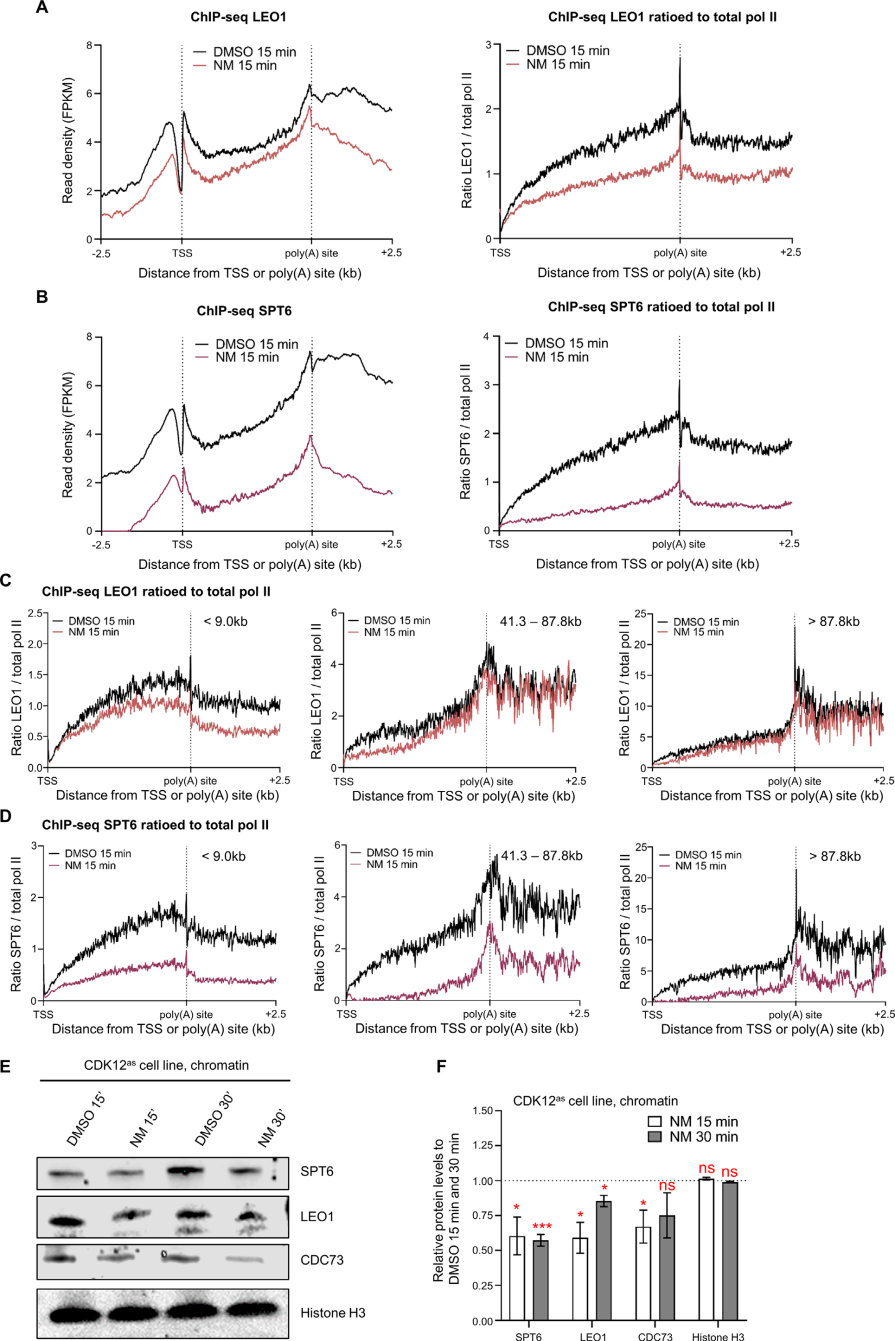
CDK12 activity is required for stable association of elongation factors with chromatin. (**A**) Meta-analysis of LEO1 subunit of the PAF1 complex (PAF1C) ChIP-seq across scaled expressed genes. (**B**) Meta-analysis of SPT6 ChIP-seq across scaled expressed genes. (**C**) Meta-analyses of LEO1 ChIP-seq ratioed to the total pol II signal before and after CDK12^as^ inhibition for 15 min across scaled expressed genes for the different length classes noted. (**D**) Meta-analyses of SPT6 ChIP-seq ratioed to the total pol II signal before and after CDK12^as^ inhibition for 15 min across scaled expressed genes for the different length classes noted. (**E**) Western blots of CDK12^as^ cell chromatin extracts. Cells are either treated with 7.5 μM 1-NM-PP1 or DMSO for 15 and 30 min as noted. The antibodies used are indicated on the right. Histone H3 was used as a loading control. (**F**) Quantitation of chromatin protein levels of SPT6, LEO1, CDC73, and histone H3 in CDK12^as^ cells relative to DMSO controls. Error bars = s.e.m. (*n* = 3 biological replicates). Statistical test: two-tailed unpaired *t* test, ns = not significant, * *P* < 0.05, *** *P* < 0.001.

Our results indicate that CDK12 is required for normal transcription elongation of most pol II-transcribed genes (Figures 1 and 2). However, the mechanism remains unclear. CDK12 activity may be required for the recruitment of elongation factors to transcribing pol II or for the stabilization of their interactions with pol II. We have therefore tested by ChIP-seq and ChIP-qPCR whether the levels of the LEO1 and CDC73 subunits of the elongation factor PAF1 complex (PAF1C) and the elongation factor SPT6 detected in chromatin are affected by CDK12^as^ inhibition (Figure 5A–C and S7). We have displayed the LEO1 and SPT6 ChIP-seq results with or without normalisation to pol II, to take the changes in pol II levels into account (Figure 5A and B). The level of LEO1 and SPT6 associated with expressed genes appears to be affected genome-wide. In addition, LEO1 appears to be selectively lost from the newly-elongating pol II as long genes (e.g. >41.3 kb) have a reduction in the ratio of LEO1 to total pol II at the 5′ end, but not at the 3′ end, where the pol II that initiated before treatment is still elongating (Figure 5C). In contrast, SPT6 is reduced more globally as a decrease in signal is also observed at the 3′end of long genes, albeit less than at the 5′ end of genes (Figure 5D). In addition, the association of CDC73 and SPT6 by ChIP-qPCR with *KPNB1*, with or without normalisation to pol II, is reduced after 15 min inhibition of CDK12^as^ (Supplementary Figure S7C). Similarly to LEO1, the level of CDC73 on *KPNB1*, which is ∼34 kb long, is decreased in the gene body but not at the 3′ end by CDK12^as^ inhibition. Western analyses also indicate that inhibition of CDK12^as^ decreases the association of SPT6, LEO1, and CDC73 with chromatin (Figure 5E and F). The loss of SPT6, LEO1, and CDC73 from chromatin is mirrored by an increase in the level of these proteins in nucleoplasm fraction after CDK12^as^ inhibition, with a particularly marked increase in nucleoplasmic SPT6 (Supplementary Figure S8A and B). These results are in line with a loss of association of these factors with chromatin, although we cannot rule out that changes in post-translation modifications are affecting antibody reactivity. Importantly, no loss of these factors from chromatin is observed when HEK293 cells are treated with NM (Supplementary Figure S8C–F). Also, the decrease on chromatin of SPT6, LEO1, and CDC73 is not due to protein degradation, as the global protein level of these elongation factors remains stable after NM treatment, both in HEK293 and CDK12^as^ cells (Supplementary Figure S8G and H). The results of immunoprecipitation of total pol II from cell extracts followed by western analyses of total pol II and SPT6 after 15 min inhibition of CDK12^as^ also suggest that SPT6 association with pol II is reduced (Supplementary Figure S9). Taken together, these results support the notion that CDK12 activity plays a key role in ensuring that the critical elongation factors PAF1C and SPT6 are part of the pol II elongation complex (19,39,40). Loss of these factors could readily explain the elongation defect that we observe after CDK12 inhibition.

It was previously shown that CDK12 is required for the recruitment of polyadenylation/transcription termination factors (17,18). To investigate further whether the loss of Ser2P and PAF1C following CDK12^as^ inhibition affects the recruitment of termination factors, we performed ChIP-seq of CPSF73, the enzyme responsible for the cleavage of the pre-mRNA at the poly(A) site (Supplementary Figure S10). We observed a general loss of CPSF73 levels after 15 min of CDK12^as^ inhibition (Supplementary Figure S10A). Importantly, the level of CPSF73 is unaffected at the 3′end of long genes (> 41.3 kb), suggesting that, similarly to LEO1 and CDC73, CPSF73 is lost from the newly-elongating pol II (Supplementary Figure S10B). We confirmed the drop in CPSF73 levels detected at the same point as newly-initiated pol II after CDK12^as^ inhibition by ChIP-qPCR on the *KPNB1* gene (Supplementary Figure S10C). These results are in agreement with the finding that Ser2P is retained relative to pol II at the 3′ end of long genes (Supplementary Figure S11). In contrast Ser5P is reduced relative to pol II at the end of long genes (Supplementary Figure S11).

## DISCUSSION

Here, we have established a protocol to rapidly and specifically inhibit CDK12 in human cells and immediately monitor changes in RNA synthesis and the occupancy of engaged pol II genome-wide. We have restricted our analysis to the first 30 min after inhibition to capture the primary effect of loss of CDK12 kinase activity. An additional benefit of our strategy is the potential to lose the activity of the kinase independently of losing the protein itself. In contrast to previous studies, our results indicate that CDK12 activity is required for efficient elongation of transcription of the vast majority of expressed pol II-dependent genes, indicating that it is a general transcription kinase, rather than playing a specific role in transcription of genes longer than 45 kb (25). Our results further suggest that CDK12 plays an increasing role in elongation after pol II has passed the CDK9-regulated early-elongation checkpoint (EEC) and entered productive elongation (Supplementary Figure S3C and D) (41). It has been shown that the pol II elongation rate increases during transcription (42) and CDK12 may therefore play a key role in pol II speeding up as it travels away from the EEC, explaining the increase in the elongation defect as pol II moves further from the TSS. Expression of long genes will therefore be disproportionately affected by an elongation defect and this will be amplified over time. This would therefore explain why CDK12 knockdown experiments show a loss of expression of long genes, in particular DDR genes. In addition, as CDK12 makes important contacts with RNA processing and elongation factors (12,18,19), the long-term loss of the protein itself may contribute to the gene-type-specific defects in the level of mRNA from long DDR genes. A recent study with CDK12^as^ HCT116 cells also indicates that CDK12 kinase activity is specifically required for transcription of long DDR genes (13). However, in this case, pol II ChIP-seq was performed after 4.5 h inhibition, which will result in a bias in the analysis towards detection of a defect on long genes.

**Figure 6.**
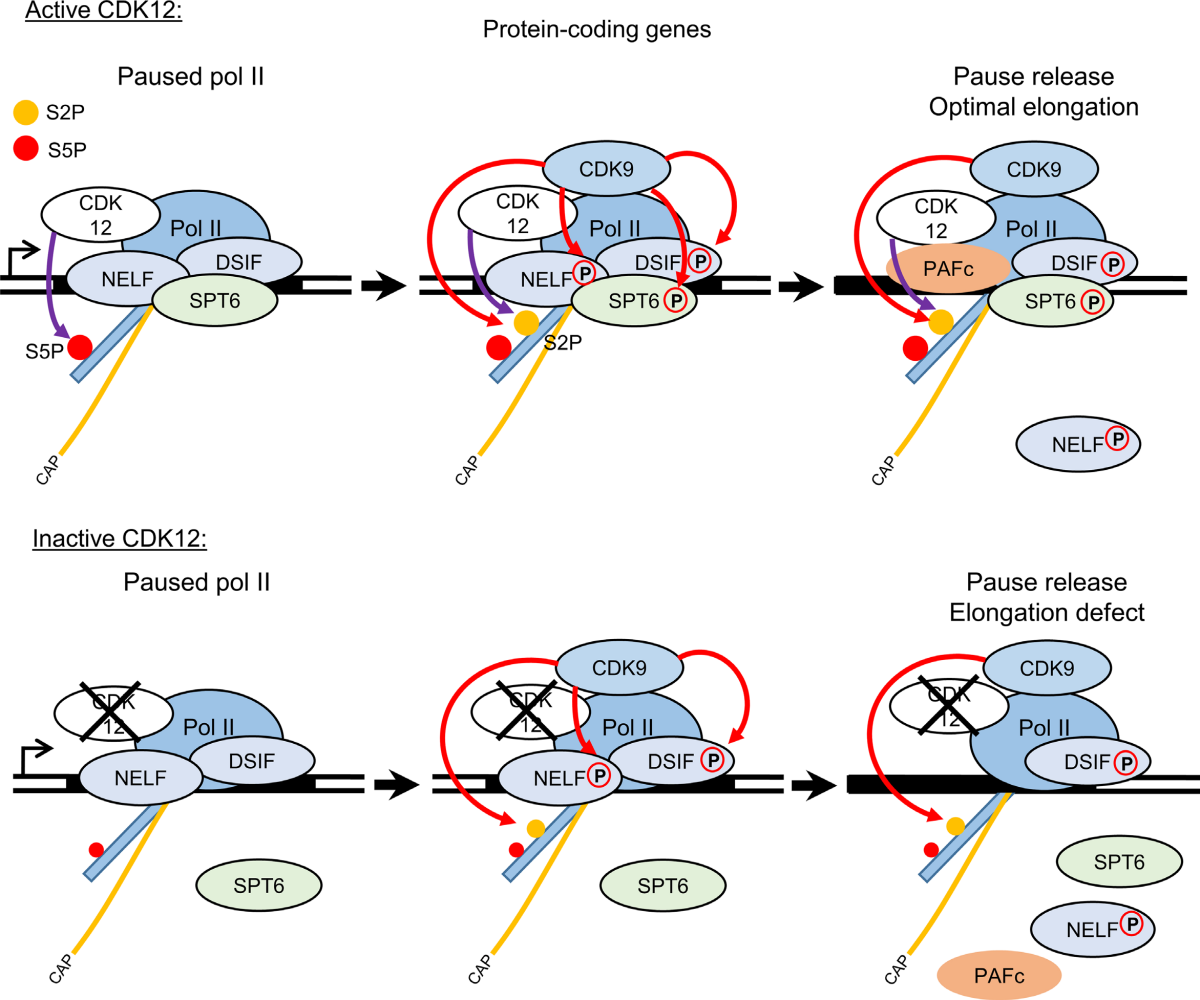
Model of CDK12 function in transcription. During the transcription cycle, CDK12 phosphorylates Ser2 and Ser5 of the pol II CTD and possibly other factors involved in transcription such as SPT6. Inhibition of CDK12 affects the pol II elongation rate by affecting the recruitment and/or stability on the chromatin of LEO1 and CDC73 from the PAF1 complex (PAF1C) and SPT6. CDK12 inhibition does not affect pol II pause release, which is mediated by CDK9. Orange line with ‘cap’: capped mRNA; orange and red dot: Ser2P and Ser5P, respectively. Red and violet arrows: phosphorylation mediated by CDK9 and CDK12, respectively.

The effect of CDK12^as^ inhibition on elongation of transcription is readily explained by the loss of association of LEO1 and CDC73, which are key components of the PAF1C elongation complex, and the elongation factor SPT6. We cannot rule out that CDK12^as^ inhibition causes rapid nuclear export of these factors or that their association with chromatin appears reduced due to epitope masking caused by changes in post-translational modifications. However, CDK12as inhibition not only causes a decrease in the level of elongation factors on chromatin but an increased level in the nucleoplasm as would be expected if CDK12 activity is required for their effective recruitment to and/or stable association with pol II. CDK12 has already been shown to interact with the PAF1 complex (19). It has also recently been shown that the CDK12/CDK13 inhibitor, THZ531, affects phosphorylation of SPT6 (25). Phosphorylation of SPT6 by CDK12 could therefore play a role in contacts between SPT6 and other components of the elongation complex, including pol II and PAF1C (40). In turn, loss of SPT6 association may further destabilize the elongation complex (43). We therefore favour the notion that CDK12 inhibition causes reduced association of elongation factors with pol II through the loss of phosphorylation of one or more of its targets. Both loss of CDK12 and inhibition of CDK12 and CDK13 by THZ531 lead to the activation of gene-internal poly(A) sites and premature termination of transcription (10,25), which may be the direct result of slower elongation as suggested (25) (Figure 6).

We also observed a loss of the polyadenylation factor CPSF73 from newly-elongating pol II (Supplementary Figure S10). It was previously shown that CDC73 can interact with CPSF73 (44) and our data support this observation as both PAF1C and CPSF73 are lost from the newly-elongating pol II. CTD Ser2P also plays a role in CPSF73 recruitment (17). At the 3′ end of long genes, the retention of CDC73 and Ser2P would therefore be sufficient to recruit CPSF73 to/stabilize its interaction with the pol II elongation complex, in the absence of CDK12 activity. As Ser5P is somewhat lost from the 3′ end of long genes, this mark is likely to be less important for PAF1C/CPSF recruitment at this point.

Notably, the effect of inhibiting CDK12 differs markedly from the effect of CDK9 inhibition, which causes a drastic loss of elongating pol II downstream of the early-elongation checkpoint (EEC) (31,45). CDK9 and CDK12 therefore play non-redundant roles in ensuring efficient transcription of pol II-dependent genes. However, these two kinases are intimately connected as CDK9 activity is required for PAF1C recruitment and PAF1C in turn recruits CDK12 (1,19). In addition, inhibition of both CDK12 and CDK9 causes premature termination close to poly(A) sites where Ser2P is peaking (45,46), indicating that the activity of both kinases is required for the correct transition between elongation and termination, through their phosphorylation of Ser2 or other targets.

CDK12 knockdown and THZ531 inhibition studies indicate that CDK12 phosphorylates Ser2 of the human pol II CTD (3,4,7), whereas *in vitro* CDK12 has robust Ser5 kinase activity (15,16). We find that detectable losses of Ser2P and Ser5P occur rapidly after CDK12^as^ inhibition, with the biggest effect on Ser5P. Relevant to this, specific inhibition of CDK12^as^ in HeLa cells results in a decrease in detection of pol II by both the H14 and H5 antibodies, which recognize Ser5P and Ser2P, respectively (14,47,48). A recent study with a CDK12^as^ HCT116 cell line observed a decrease in Ser5P but not of Ser2P following CDK12 inhibition (13). We have shown here that association of a Ser5P-binding capping factor with genes is affected by CDK12^as^ inhibition. Our results therefore support the notion that CDK12 is a major Ser5 kinase. However, based on previous findings, we would have expected inhibition of CDK12^as^ to cause a greater loss of Ser2P. Thus, in at least some human cells, the Ser2 kinase activity of CDK12 may be somewhat redundant with other Ser2 kinases, such as CDK9.

In conclusion, we have shown that inhibition of CDK12 causes a genome-wide pol II defect in pol II velocity and processivity and loss of PAF1C components and SPT6 from the newly-elongating pol II. Our findings also indicate that CDK12 is both a CTD Ser2 and Ser5 kinase *in vivo*. It remains to be determined whether CDK12 function in transcription elongation is mediated through pol II CTD phosphorylation and/or through phosphorylation of other targets such as SPT6.

## DATA AVAILABILITY

mNET-seq and ChIP-seq data have been deposited in GEO under accession number GSE115290. TT-seq data have been deposited in GEO under accession number GSE133156.

## Supplementary Material

gkaa514_Supplemental_File
