# Supplementary material for: CDK12 globally stimulates RNA polymerase II transcription elongation and carboxyl-terminal domain phosphorylation
Source: Nucleic Acids Res. 2020 Jun 17;48(14):7712–27. doi: 10.1093/nar/gkaa514 (PMC7641311; doi:10.1093/nar/gkaa514)
Supplement: gkaa514_Supplemental_File [file gkaa514_supplemental_file.pdf]

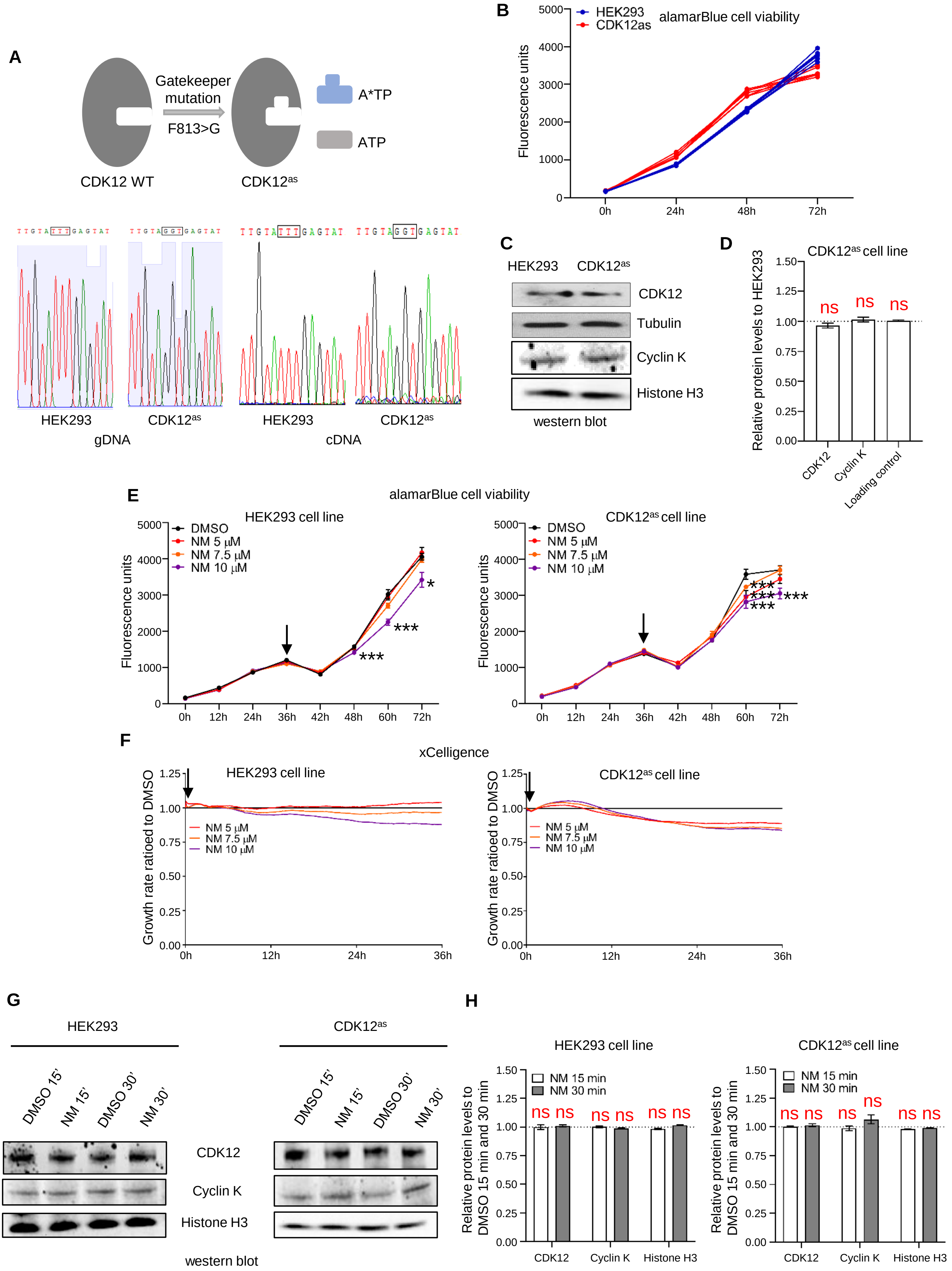

Supplementary Figure 1

Supplementary Figure 1. Characterization of the $CDK12^{as}$ HEK293 cell line.

A. Cartoon depicting the creation of $CDK12^{as}$ by altering the 'gate-keeper' residue in the kinase active site (top). CRISPR/Cas9 genome engineering was used to mutate F (TTT) to G (GGT) (bottom). Sanger sequencing tracks of gDNA and cDNA from CDK12 WT HEK293 control and $CDK12^{as}$ are shown. **B.** Time-course of the growth of viable HEK293 and $CDK12^{as}$ cells after seeding, as measured using alamarBlue HS. n = 6 biological replicates. **C.** Western blots of parental HEK293, and $CDK12^{as}$ cell whole cell extracts with the antibodies noted at the right. Tubulin and histone H3 were used as loading controls. **D.** Relative protein levels of CDK12, Cyclin K, and loading controls in $CDK12^{as}$ cells compared to HEK293 cells. Error bars = s.e.m. (n = 2 biological replicates). Statistical test: two-tailed unpaired t test, ns = not significant. **E.** Time-course of the growth of viable HEK293 and $CDK12^{as}$ cells after seeding using alamarBlue HS. Error bars= s.e.m. (n = 6 biological replicates). Statistical test: two-tailed unpaired t test, *** $p < 0.001$. The noted concentrations of NM were added at the 36 hours' time point as indicated by the arrows. **F.** Time-course of the growth of the HEK293 and $CDK12^{as}$ cells measured by xCelligence (n = 6 biological replicates). NM was added 36 hours after seeding, indicated as the 0 hour time point on the figure and indicated by the arrows. **G.** Western blots of parental HEK293, and $CDK12^{as}$ cell whole cell extracts treated with DMSO or 7.5 μM NM for 15 or 30 minutes with the antibodies noted in the middle. Histone H3 was used as a loading control. **H.** Quantitation of protein levels of CDK12, Cyclin K, and histone H3 in HEK293 and $CDK12^{as}$ cells after NM treatment relative to the control. Error bars= s.e.m. (n = 3 biological replicates). Statistical test: two-tailed unpaired t test, ns = not significant.

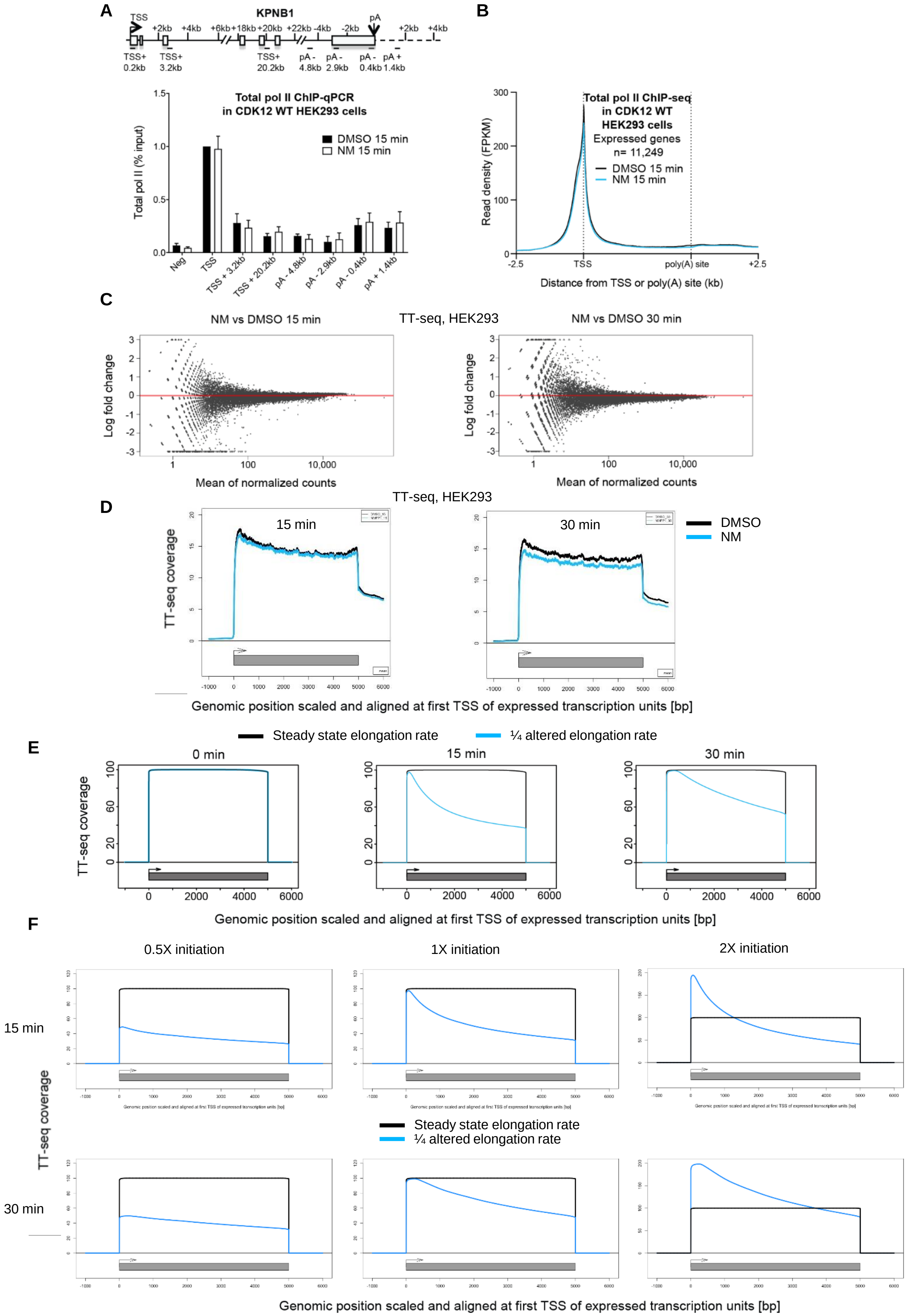

Supplementary Figure 2

Supplementary Figure 2. Characterization of the CDK12^{as} HEK293 cell line.

A. ChIP-qPCR of pol II on *KPNB1* after treatment of HEK293 cells with DMSO or 7.5 μM NM for 15 minutes. A schematic of *KPNB1* is shown above. **B.** Meta-analysis of Pol II ChIP-seq of scaled expressed genes performed on the parental HEK293 cells treated with DMSO (black) or with 7.5 μM NM (blue) for 15 minutes. **C.** Log fold change upon 7.5 μM NM treatment for 15 min (left) 30 min (right) versus mean of normalized counts across replicates in parental HEK293 cells. **D.** Meta-analysis of TT-seq data of expressed genes after treatment of HEK293 cells with DMSO (black) or 7.5 μM NM (blue) for 15 or 30 minutes. **E.** Simulated metagene profiles of expected RNA synthesis (TT-seq signal) in steady-state (left) and upon changes in elongation rate for 15 minutes (middle) and 30 minutes (right). **F.** Simulated metagene profiles of expected RNA synthesis (TT-seq signal) upon changes in initiation frequencies, 0.5X (left), 1X (middle), and 2X (right), and elongation rate for 15 minutes (top) and 30 minutes (bottom). In comparison to the steady state elongation rate profile (black), altered ¼ elongation rate profile (blue) shows lower coverage of different magnitude for above stated initiation frequencies. With an initiation frequency 2X (right) the altered elongation rate profile (blue) shows a higher coverage close to the transcription start site which decreases along the gene body for the given time window.

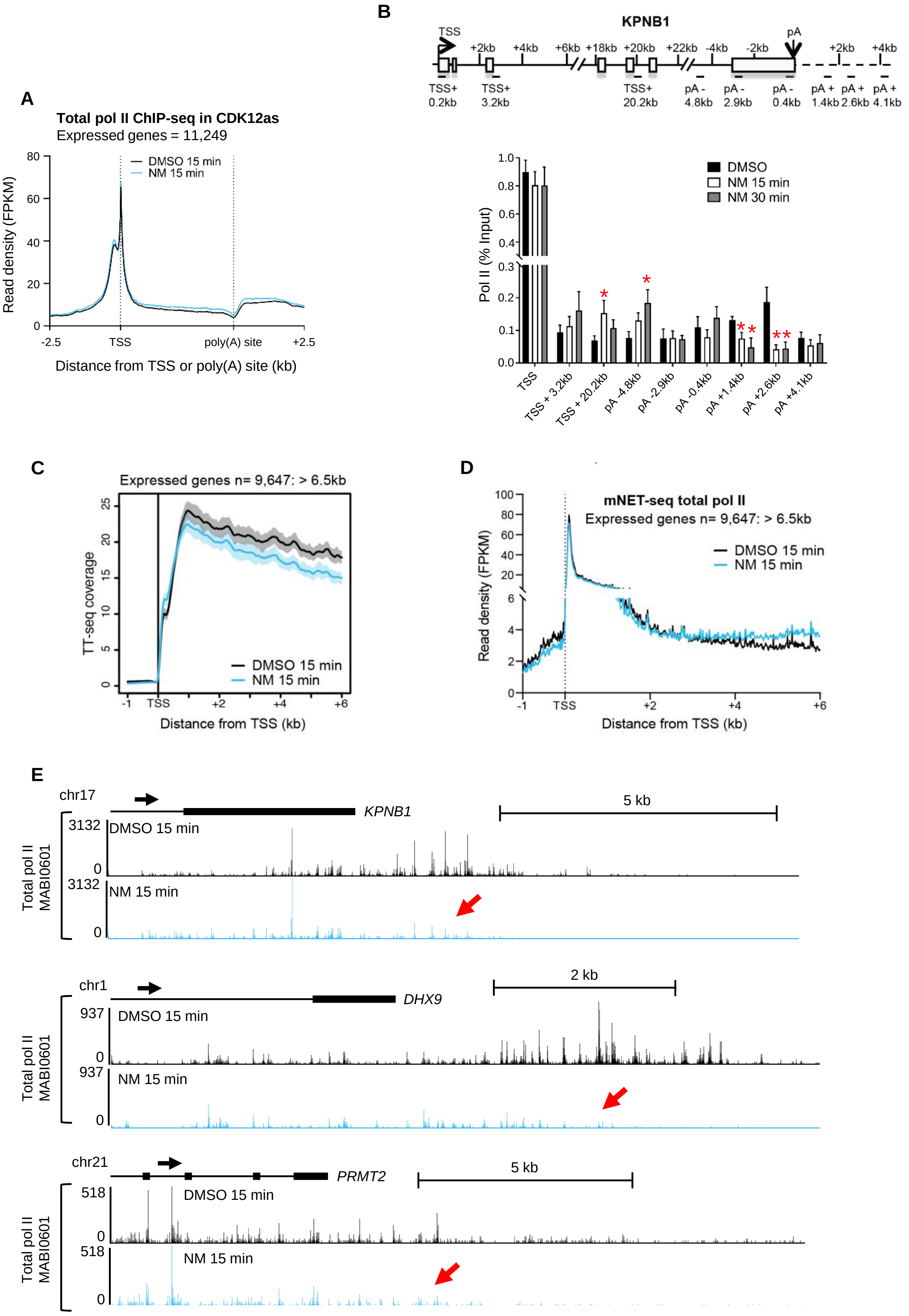

Supplementary Figure 3

Supplementary Figure 3. CDK12 inhibition affects elongation of transcription beyond the EEC.

A. Meta-analysis of Pol II ChIP-seq of scaled expressed genes performed on the CDK12^{as} cells treated with DMSO (black) or with 7.5 µM NM (blue) for 15 minutes. **B.** ChIP-qPCR of pol II on *KPNB1* after treatment of CDK12^{as} cells with DMSO or 7.5 µM NM for 15 or 30 minutes. Error bars= s.e.m. (n = 3 biological replicates). Asterisks indicate statistical significance (* $p < 0.05$), based on unpaired, two-tailed Student's t test. A schematic of *KPNB1* is shown on the top **C.** Meta-analysis of TT-seq data for the first 6 kb of expressed genes > 6.5 kb in length after treatment of CDK12^{as} cells with DMSO (black) or 7.5 µM NM (blue) for 15 minutes. **D.** Meta-analysis of mNET-seq data for the first 6kb of expressed genes > 6.5 kb in length after treatment of CDK12^{as} cells with DMSO (black) or 7.5 µM NM (blue) for 15 minutes. **E.** Total pol II mNET-seq profiles across the 3'end of *KPNB1*, *DHX9*, and *PRMT2*.

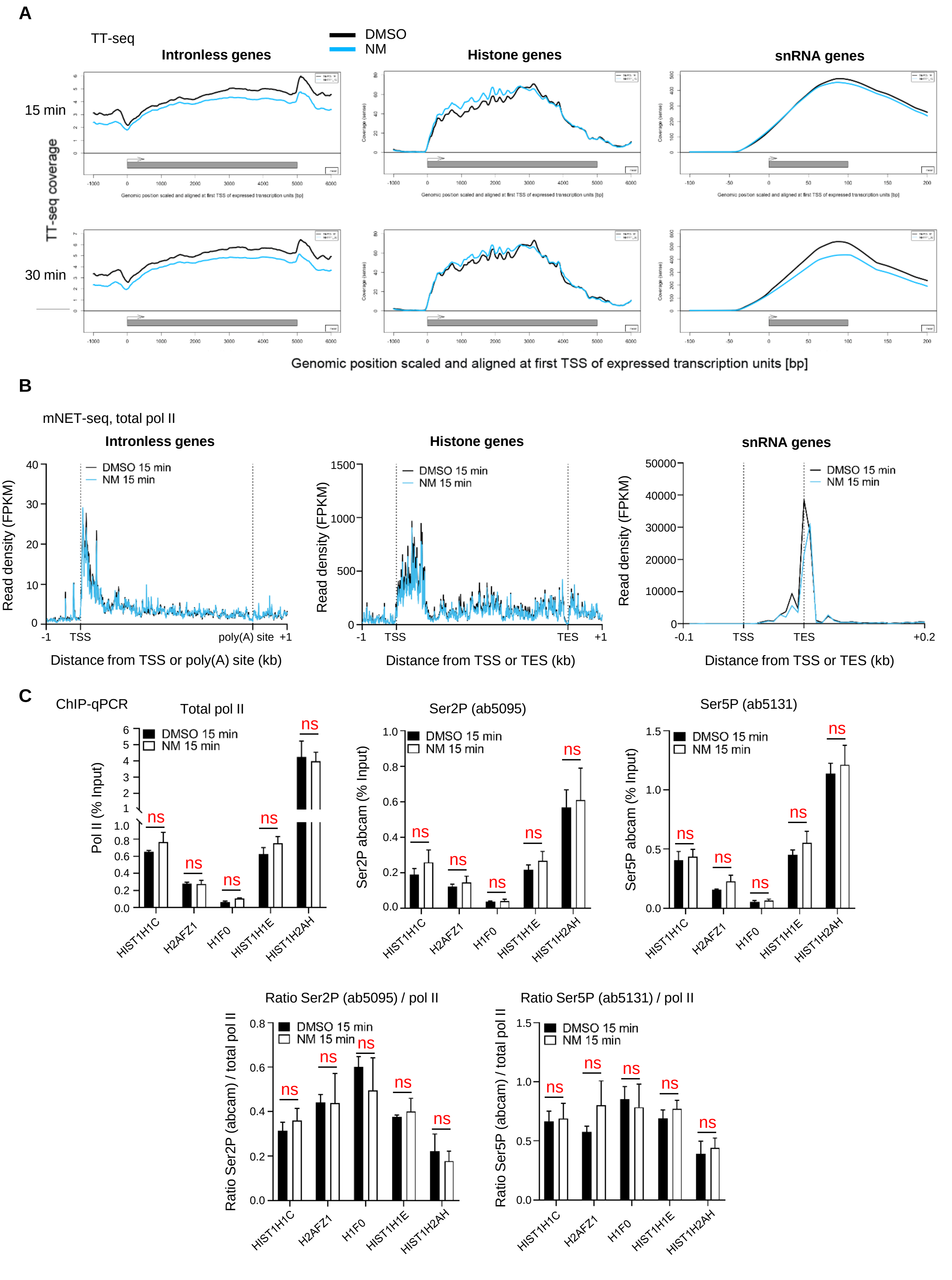

Supplementary Figure 4

Supplementary Figure 4. CDK12 inhibition does not affect transcription of histone genes.

A. Meta-analysis of TT-seq data of intronless, histone, and snRNA genes after treatment of $CDK12^{as}$ cells with DMSO (black) or 7.5 μM NM (blue) for 15 or 30 minutes. **B.** Meta-analysis of total pol II mNET-seq data of intronless, histone, and snRNA genes after treatment of $CDK12^{as}$ cells with DMSO (black) or 7.5 μM NM (blue) for 15 minutes. **C.** ChIP-qPCR of pol II, Ser2P (ab5095), and Ser5P (ab5131) on the gene body of five different histone genes after treatment of $CDK12^{as}$ cells with DMSO or 7.5 μM NM for 15 minutes. Error bars= s.e.m. (n = 3 biological replicates). Asterisks indicate statistical significance (ns: not significant), based on unpaired, two-tailed Student's t test.

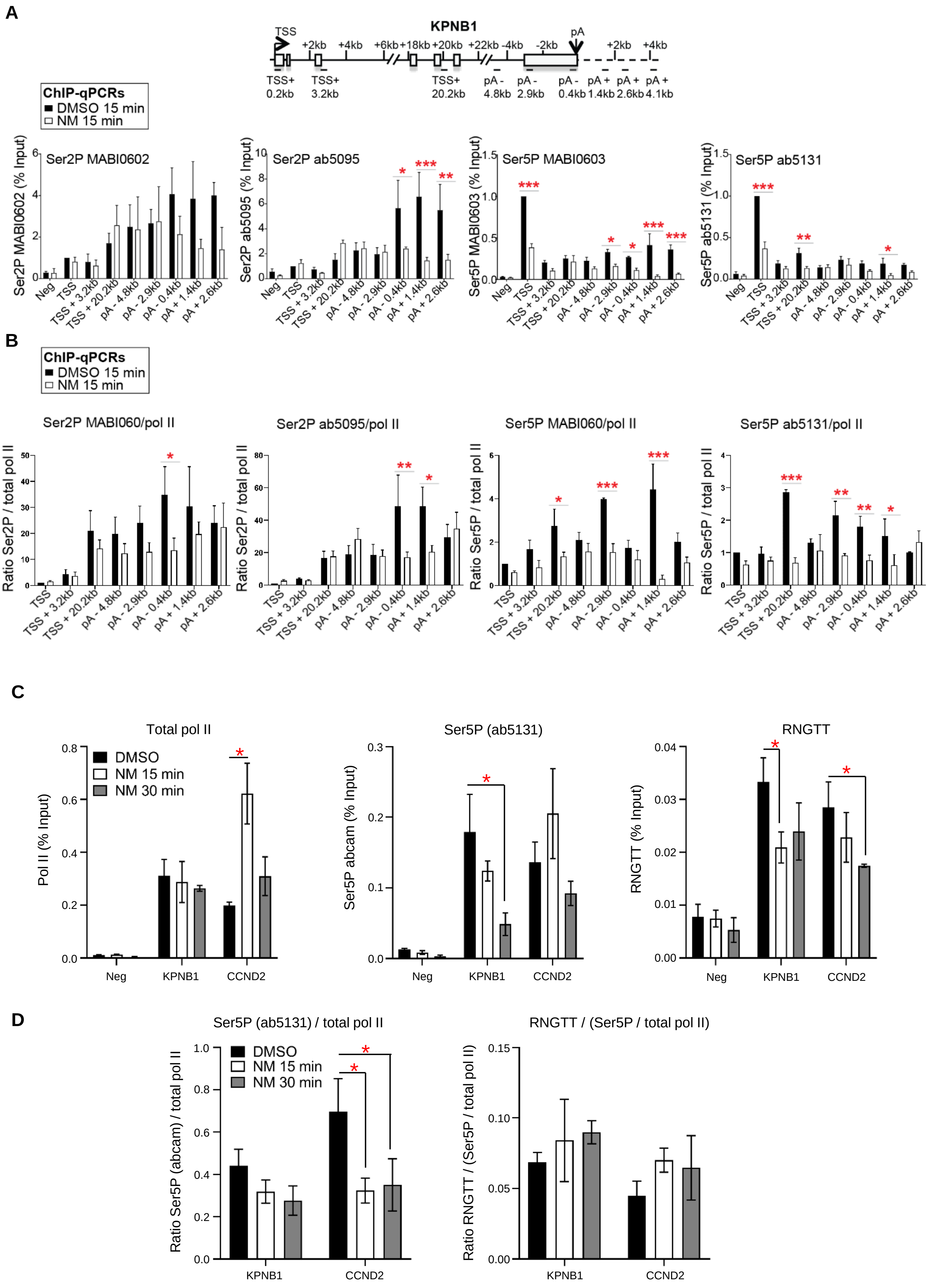

Supplementary Figure 5

Supplementary Figure 5. CDK12 regulates phosphorylation of the CTD of engaged pol II.

A. ChIP-qPCR of Ser2P and Ser5P after treatment of $CDK12^{as}$ cells with DMSO or 7.5 μM NM for 15 minutes. Error bars= s.e.m. (n = 3 biological replicates). Asterisks indicate statistical significance (* $p < 0.05$; ** $p < 0.01$; *** $p < 0.001$), based on unpaired, two-tailed Student's t test. A schematic of *KPNB1* is shown above. **B.** Ratio of the ChIP-qPCR for Ser2P and Ser5P to pol II after treatment of $CDK12^{as}$ cells with DMSO or 7.5 μM NM for 15 minutes. Error bars= s.e.m. (n = 3 biological replicates). Asterisks indicate statistical significance (* $p < 0.05$; ** $p < 0.01$; *** $p < 0.001$), based on unpaired, two-tailed Student's t test. **C.** ChIP-qPCR of total pol II, Ser5P, and RNGTT on the TSS of *KPNB1* and *CCND2* after treatment of $CDK12^{as}$ cells with DMSO or 7.5 μM NM for 15 or 30 minutes. Error bars= s.e.m. (n = 3 biological replicates). Asterisks indicate statistical significance (* $p < 0.05$), based on unpaired, two-tailed Student's t test. **D.** Ratio of the ChIP-qPCR of Ser5P to pol II or of RNGTT ratioed to Ser5P/pol II after treatment of $CDK12^{as}$ cells with DMSO or 7.5 μM NM for 15 or 30 minutes. Error bars = s.e.m. (n = 3 biological replicates). Asterisks indicate statistical significance (* $p < 0.05$), based on unpaired, two-tailed Student's t test.

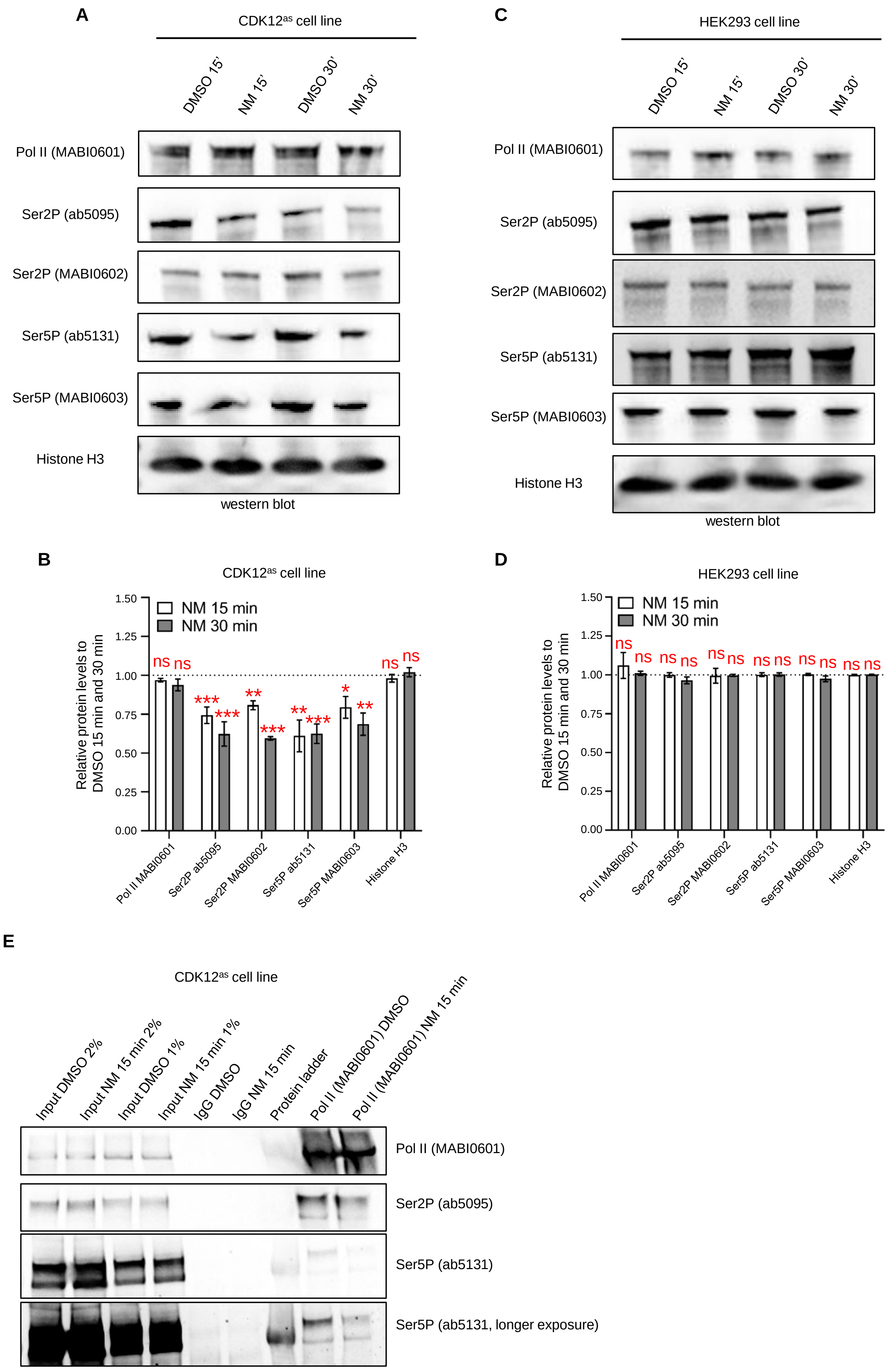

Supplementary Figure 6

Supplementary Figure 6. CDK12^{as} inhibition causes changes to CTD phosphorylation.

A. Western blots of CDK12^{as} cell chromatin extracts. Cells are either treated with 7.5 μM 1-NM-PP1 or DMSO for 15 and 30 minutes as noted. The antibodies used are indicated on the left. Histone H3 was used as a loading control. **B.** Quantitation of protein levels of total pol II, Ser2P, Ser5P and histone H3 in CDK12^{as} cells relative to DMSO controls. Error bars = s.e.m. (n = 3 biological replicates). Statistical test: two-tailed unpaired t test, ns = not significant, * $p < 0.05$, ** $p < 0.01$, *** $p < 0.001$. **C.** Western blots of chromatin extracts of DMSO-treated and NM-treated parental HEK293 cells with the antibodies noted on the left. Histone H3 was used as a loading control. **D.** Quantitation of protein levels of total pol II, Ser2P, Ser5P and histone H3 in HEK293 cells relative to DMSO controls. Error bars = s.e.m. (n = 3 biological replicates). Statistical test: two-tailed unpaired t test, ns = not significant. **E.** Co-immunoprepication of total pol II (MABI0601) followed by western blot with total pol II (MABI0601), Ser2P (ab5095), and Ser5P (ab5131) antibodies.

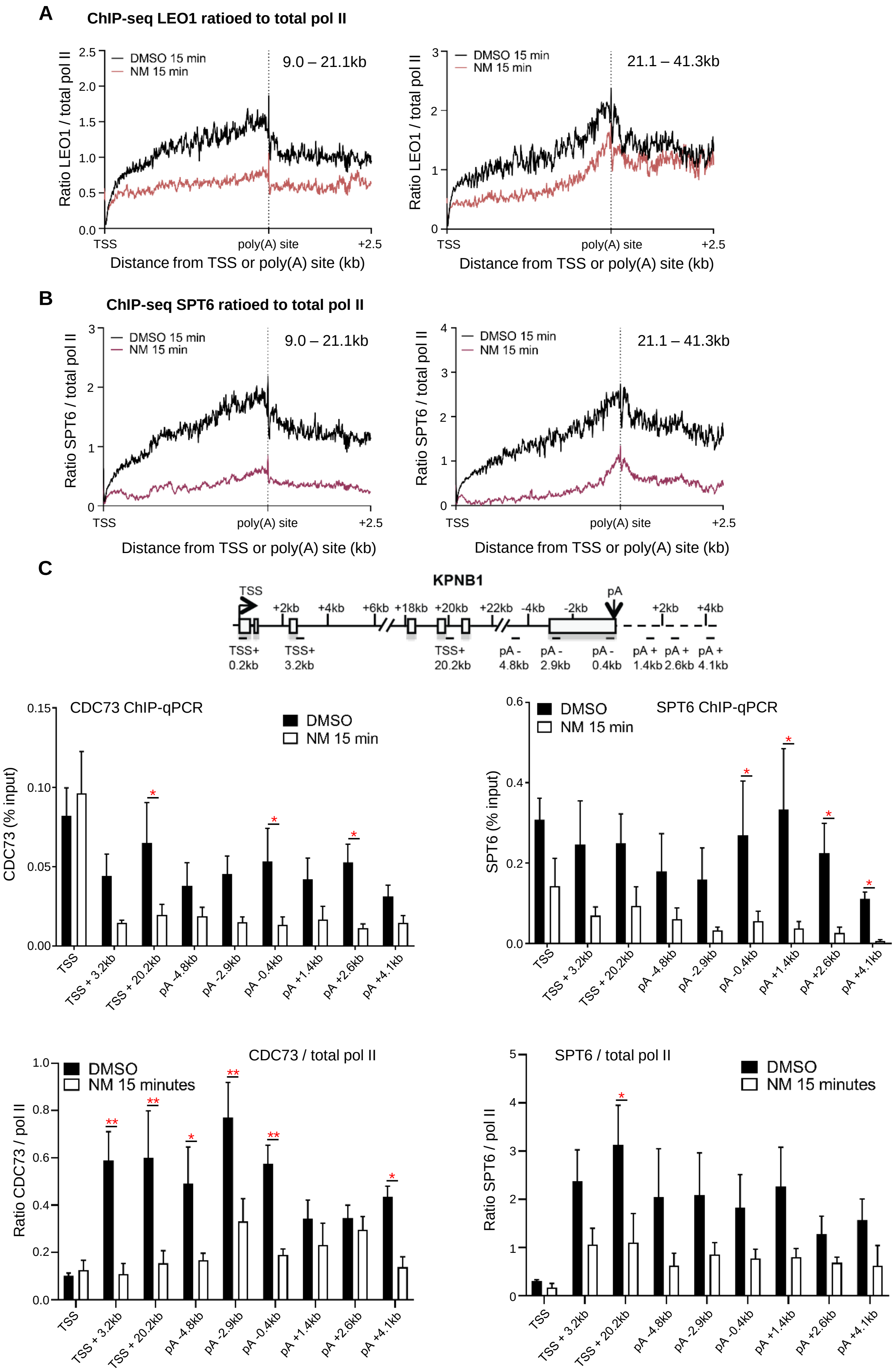

Supplementary Figure 7

Supplementary Figure 7. CDK12 activity is required for stable association of elongation factors with chromatin.

A. Meta-analyses of LEO1 ChIP-seq ratioed to the total pol II signal across scaled expressed genes for the different length classes noted. **B.** Meta-analyses of SPT6 ChIP-seq ratioed to the total pol II signal across scaled expressed genes for the different length classes noted. **C.** CDC73 and SPT6 ChIP-qPCR unratioed (top) or ratioed to pol II (bottom) across *KPNB1* gene after treatment of $CDK12^{as}$ cells with DMSO or 7.5 μM NM for 15 minutes. Error bars = s.e.m. (n = 4 biological replicates). Asterisks indicate statistical significance (* $p < 0.05$, ** $p < 0.01$), based on unpaired, two-tailed Student's t test.

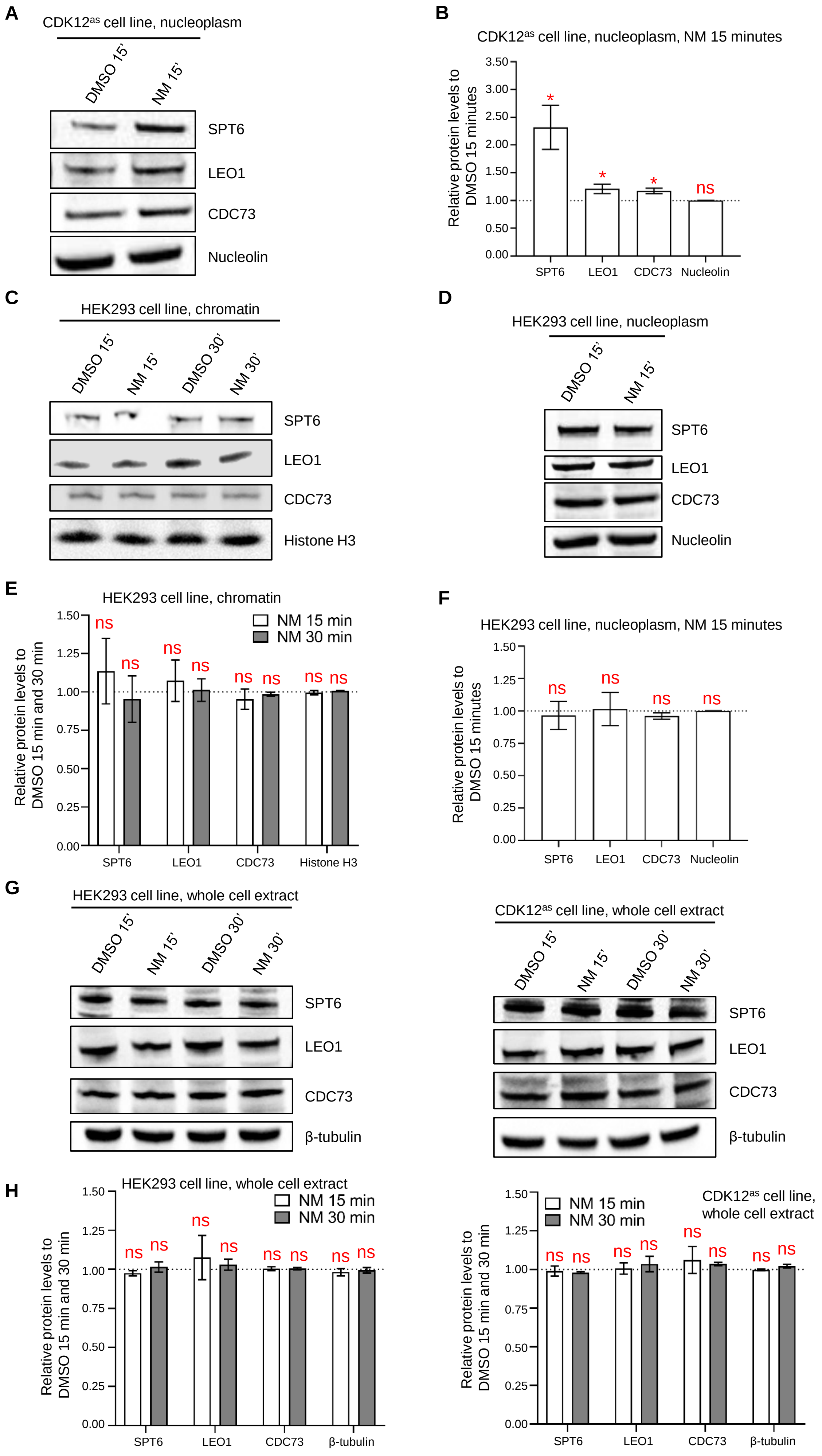

Supplementary Figure 8

Supplementary Figure 8. CDK12 activity is required for stable association of elongation factors with chromatin.

A. Western blots of CDK12^{as} cell nucleoplasmic extracts. Cells are either treated with 7.5 μM 1-NM-PP1 or DMSO for 15 minutes as noted. The antibodies used are indicated on the right. Nucleolin was used as a loading control. **B.** Quantitation of nucleoplasmic protein levels of SPT6, LEO1, CDC73, and nucleolin in CDK12^{as} cells relative to DMSO controls. Error bars = s.e.m. (n = 3 biological replicates). Statistical test: two-tailed unpaired t test, ns = not significant, * $p < 0.05$, **C.** Western blots of HEK293 cell chromatin extracts. Cells are either treated with 7.5 μM 1-NM-PP1 or DMSO for 15 and 30 minutes as noted. The antibodies used are indicated on the right. Histone H3 was used as a loading control. **D.** Western blots of HEK293 cell nucleoplasmic extracts. Cells are either treated with 7.5 μM 1-NM-PP1 or DMSO for 15 minutes as noted. The antibodies used are indicated on the right. Nucleolin was used as a loading control. **E.** Quantitation of chromatin protein levels of SPT6, LEO1, CDC73, and histone H3 in HEK293 cells relative to DMSO controls. Error bars = s.e.m. (n = 3 biological replicates). Statistical test: two-tailed unpaired t test, ns = not significant. **F.** Quantitation of nucleoplamsic protein levels of SPT6, LEO1, CDC73, and nucleolin in HEK293 cells relative to DMSO controls. Error bars = s.e.m. (n = 3 biological replicates). Statistical test: two-tailed unpaired t test, ns = not significant. **G.** Western blots of HEK293 (left) or CDK12^{as} (right) cell whole cell extracts. Cells are either treated with 7.5 μM 1-NM-PP1 or DMSO for 15 and 30 minutes as noted. The antibodies used are indicated in the middle. β-tubulin was used as a loading control. **H.** Quantitation of whole cell extract protein levels of SPT6, LEO1, CDC73, and β-tubulin in HEK293 (left) or CDK12^{as} (right) cells relative to DMSO controls. Error bars = s.e.m. (n = 3 biological replicates). Statistical test: two-tailed unpaired t test, ns = not significant.

A

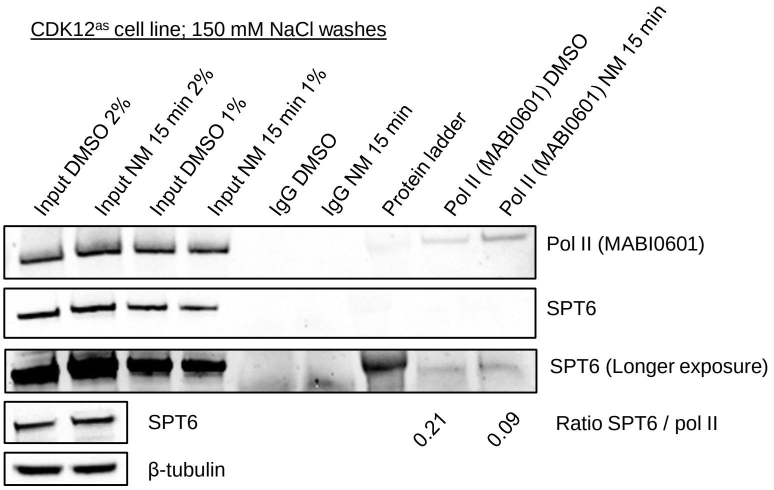

B

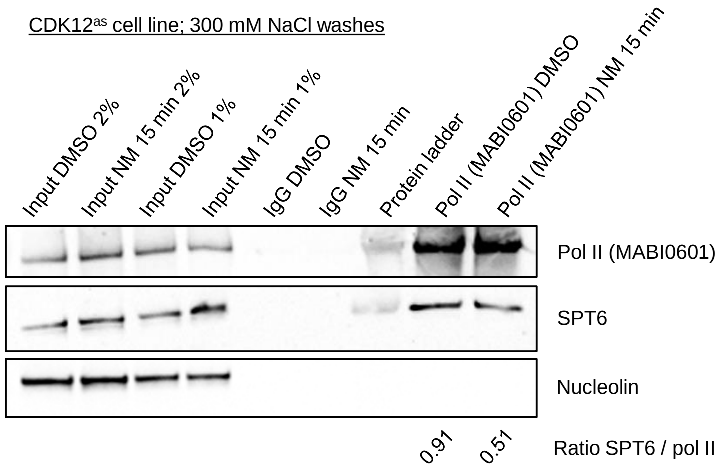

Supplementary Figure 9

Supplementary Figure 9. CDK12 inhibition causes loss of detectable SPT6 associated with pol II.

Co-immunoprecipitation of total pol II (MABI0601) from $CDK12^{as}$ cells treated with DMSO or 7.5 µM NM for 15 minutes and washed with 150 mM (**A/**) or 300 mM (**B/**) NaCl followed by western blot with total pol II (MABI0601) and SPT6 antibodies. β-tubulin and nucleolin are used as loading controls. Ratios of SPT6 to total pol II are indicated below each western blot.

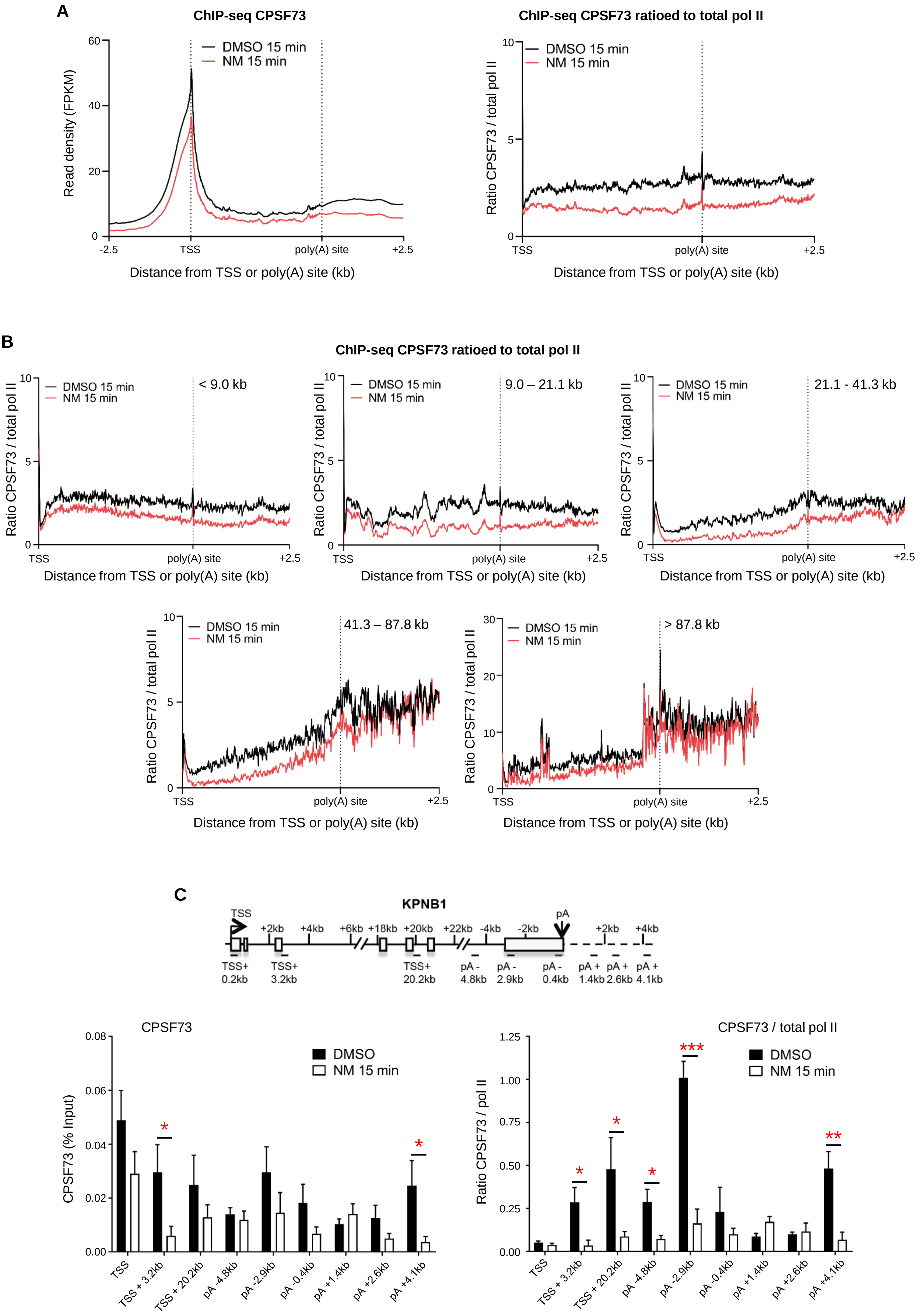

Supplementary Figure 10

Supplementary Figure 10. CDK12 activity is required for stable association of the polyadenylation factor CPSF73.

A. Meta-analyses of scaled expressed genes of ChIP-seq for CPSF73 with and without normalization to pol II after treatment of $CDK12^{as}$ cells with DMSO (black) or 7.5µM NM (red) for 15 minutes **B.** Metagene analyses for the different length classes noted comparing the CPSF73 signal ratioed to the total pol II mNET-seq signal before and after $CDK12^{as}$ inhibition for 15 minutes. **C.** CPSF73 ChIP-qPCR unratioed (left) or ratioed to pol II (right) across *KPNB1* gene after treatment of $CDK12^{as}$ cells with DMSO or 7.5 µM NM for 15 minutes. Error bars = s.e.m. (n = 3 biological replicates). Asterisks indicate statistical significance (* $p < 0.05$, ** $p < 0.01$, *** $p < 0.001$), based on unpaired, two-tailed Student's t test.

A

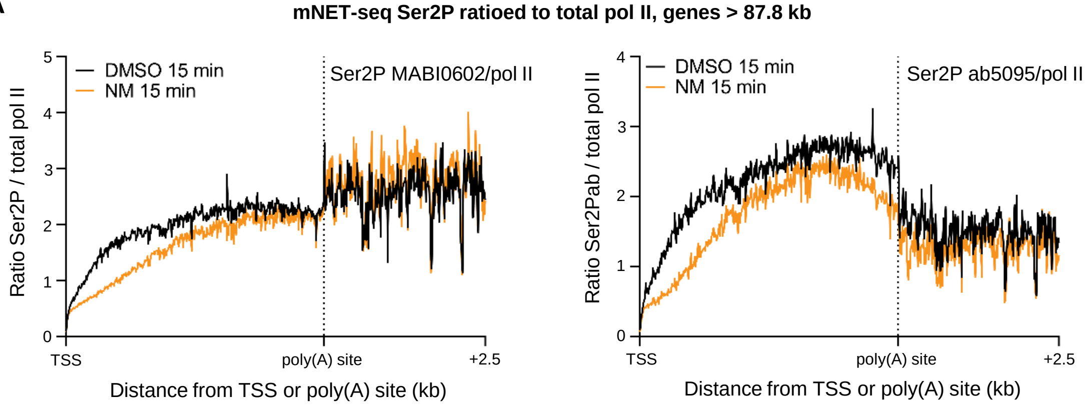

B

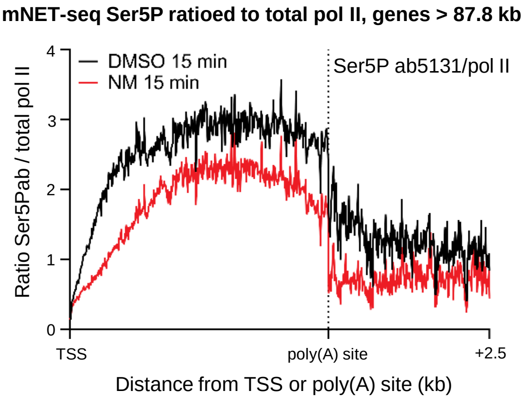

Supplementary Figure 11

Supplementary Figure 11. Ser2P is unaffected at the 3’end of long genes.

A. Meta-analyses of mNET-seq for Ser2P (MABI0602 and ab5095) ratioed to pol II across genes > 87.8 kb after treatment of $CDK12^{as}$ cells with DMSO (black) or 7.5µM NM (orange) for 15 minutes. **B.** Meta-analyses of mNET-seq for Ser5P (ab5131) ratioed to pol II across genes > 87.8 kb after treatment of $CDK12^{as}$ cells with DMSO (black) or 7.5µM NM (red) for 15 minutes.
